# Targeted DNA ADP-ribosylation triggers templated repair in bacteria and base mutagenesis in eukaryotes

**DOI:** 10.1038/s41587-025-02802-w

**Published:** 2025-09-04

**Authors:** Darshana Gupta, Constantinos Patinios, Harris V. Bassett, Anuja Kibe, Scott P. Collins, Charlotte Kamm, Yanyan Wang, Chengsong Zhao, Katie Vollen, Christophe Toussaint, Irene Calvin, Grégoire Cullot, Eric J. Aird, Kathryn M. Polkoff, Thuan Phu Nguyen-Vo, Angela Migur, Friso Schut, Ibrahim S. Al’Abri, Tatjana Achmedov, Alessandro Del Re, Jacob E. Corn, Antoine-Emmanuel Saliba, Nathan Crook, Anna N. Stepanova, Jose M. Alonso, Chase L. Beisel

**Affiliations:** 1 Helmholtz Institute for RNA-based Infection Research (HIRI), Helmholtz Centre for Infection Research (HZI), Würzburg, Germany; 2 Department of Chemical and Biomolecular Engineering, North Carolina State University, Raleigh, NC USA; 3 Department of Plant and Microbial Biology, North Carolina State University, Raleigh, NC USA; 4 Institute of Molecular Health Sciences, Department of Biology, Swiss Federal Institute of Technology (ETH) Zurich, Zurich, Switzerland; 5 Institute of Molecular Infection Biology, University of Würzburg, Würzburg, Germany; 6 Medical Faculty, University of Würzburg, Würzburg, Germany

**Keywords:** Synthetic biology, Genetic engineering

## Abstract

Base editors create precise genomic edits by directing nucleobase deamination or removal without inducing double-stranded DNA breaks. However, a vast chemical space of other DNA modifications remains to be explored for genome editing. Here we harness the bacterial antiphage toxin DarT2 to append ADP-ribosyl moieties to DNA, unlocking distinct editing outcomes in bacteria versus eukaryotes. Fusing an attenuated DarT2 to a Cas9 nickase, we program site-specific ADP-ribosylation of thymines within a target DNA sequence. In tested bacteria, targeting drives homologous recombination, offering flexible and scar-free genome editing without base replacement or counterselection. In tested yeast, plant and human cells, targeting drives substitution of the modified thymine to adenine or a mixture of adenine and cytosine with limited insertions or deletions, offering edits inaccessible to current base editors. Altogether, our approach, called append editing, leverages the addition of chemical moieties to DNA to expand current modalities for precision gene editing.

## Abstract (web summary)

Append editing of ADP-ribosyl to thymine is used for precise modifications in bacteria and eukaryotes.

## Main

In the expanding field of genome editing, targeting chemical modifications to a specific DNA sequence offers an effective way to create precise genomic edits without relying on double-stranded (ds)DNA breaks^1–3^. These modifications are installed at selected sites by base editors (BEs) comprising an enzymatic DNA domain and a programmable DNA binding protein. After the BE acts on recognized bases within a selected target site, the modified bases then change identity, resulting in a permanent genetic substitution. As this process does not actively generate dsDNA breaks at the target site, unintended and possibly harmful genetic alterations such as random insertions or deletions (indels), chromosomal abnormalities or chromothripsis are avoided^1,4^. To date, BEs have been applied in all three domains of life^5,6^ including DNA-containing organelles such as mitochondria^7^; they can convert each of the four bases^6^ and have recently entered clinical use^8^.

Within these advances, BEs have consistently relied on DNA deaminases to remove an amino group, changing the base’s perceived identity, or on DNA glycosylases to remove the entire base, driving the base’s replacement through base excision repair^2,9^. While such ‘subtractive’ DNA modifications represent powerful means to elicit precise gene edits, what remains unexplored is the impact of ‘additive’ DNA modifications. Extensive work in DNA repair has shown that appended chemical moieties can elicit diverse DNA repair pathways, such as homologous recombination, translesion synthesis, nucleotide excision repair or Fanconi anemia repair, extending well beyond base excision repair^10–12^. However, the programmable addition of chemical moieties to DNA for gene editing remains to be explored.

**Fig. 1:**
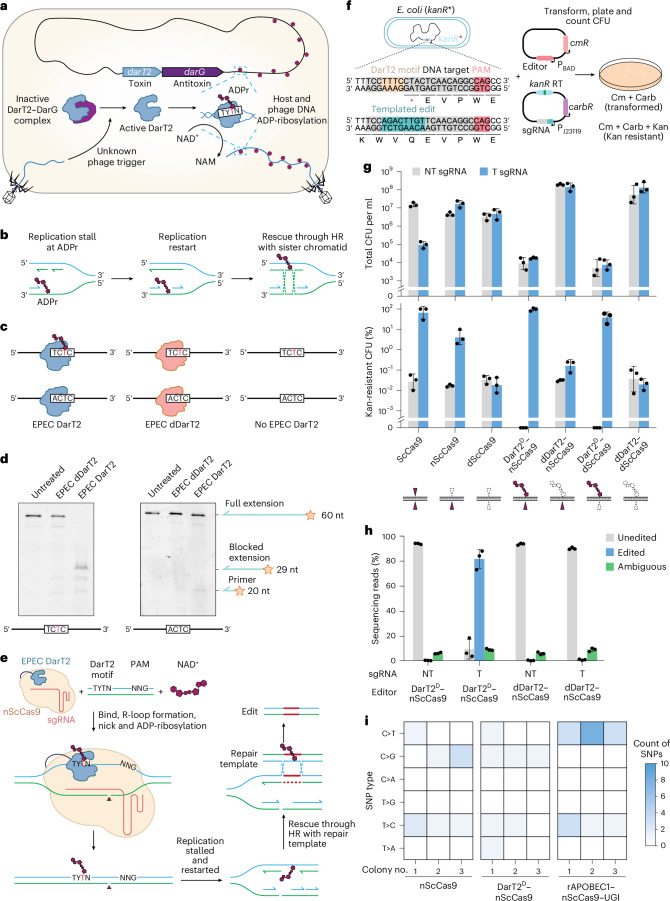
Targeted DNA ADP-ribosylation drives template-mediated homologous recombination in *E.* *coli.* **a**, Role of the bacterial DarT2 toxin in antiphage immunity. NAM, niacinamide. **b**, Conceptualized impact and resolution of DNA ADP-ribosylation on DNA replication in *E.* *coli*^17^. **c**, Experimental setup for the in vitro polymerase-blocking assay. EPEC DarT2 recognizes the 5′-TCTC-3′ but not the 5′-ACTC-3′ motif. **d**, Impact of DNA ADP-ribosylation by DarT2 on DNA polymerase extension in vitro. Gel images are representative of two independent experiments (additional controls in Extended Data Fig. 1). **e**, Configuration of the append editor using DarT2. The editor combines ScCas9 mutated to nick the target DNA strand and a fused DarT2 that ADP-ribosylates the nontarget DNA strand displaced as part of R-loop formation. This combination is predicted to drive homologous recombination with a provided repair template (RT). HR, homologous recombination. **f**, Experimental setup for reverting a prematurely terminated kanamycin resistance gene (kanR*) in *E.* *coli*. The chromosomally integrated gene contains a premature stop codon that is reverted as part of homologous recombination, thus conferring kanamycin resistance. Cm, chloramphenicol; Carb, carbenicillin; Kan, kanamycin. **g**, Impact of programmable DNA ADP-ribosylation on cell viability and kanamycin resistance frequency. Bars and error bars represent the geometric mean and geometric s.d. of three independent experiments started from separate transformations. Dots represent individual measurements. CFU, colony-forming units. Bottom, cartoons designate whether a given DNA strand is unaltered, nicked or ADP-ribosylated. **h**, Amplicon sequencing of the kanR* target site from batch cultures. Bars and error bars represent the mean and s.d. of three independent experiments starting from separate transformations. Dots represent individual measurements. **i**, Genome-wide profiling of off-target edits. The indicated editor was expressed with an NT sgRNA in the absence of an RT. More information on the identified edits can be found in Supplementary Table 1. Whole-genome sequencing was performed on genomic DNA extracted from cultures beginning with an individual colony. Both strands are considered for a given edit (for example, T > A and A > T are combined). sgRNA, single-guide RNA; SNP, single-nucleotide polymorphism. Source data

One promising starting point derives from the DNA ADP-ribosyltransferase protein DarT2 (ref. ^13^). DarT2 is part of the DarT2/DarG toxin–antitoxin system recently associated with a growing collection of antiphage defenses (Fig. 1a)^14^. As the system’s toxin, DarT2 appends a single ADP-ribosyl (ADPr) moiety to the N3 position of thymine in single-stranded (ss)DNA using the metabolic cofactor NAD^+^ as a substrate^15^. The antitoxin DarG protein catalytically removes the appended ADPr moiety and also serves as a DNA mimic that binds DarT2 (ref. ^16^). During a phage infection, DarG is inactivated through an unknown mechanism and DarT2 begins ADP-ribosylating DNA within the bacteriophage and host genome^14^. An appended ADPr moiety interferes with DNA replication, which can block bacteriophage replication and induce cellular growth arrest. In *Escherichia coli*, growth arrest can be partially relieved through bypass by RecF-mediated homologous recombination with the sister chromatid followed by removal through nucleotide excision repair (Fig. 1b)^17^. Critically, this mode of repair contrasts with traditional base editing in this bacterium^18,19^, suggesting that the installation of an ADPr moiety could unlock distinct types of genome edits. Here, we explore such an approach, which we call append editing. As we append an ADPr moiety to thymine, the approach can be abbreviated as ADPr-T append editing, or ADPr-TAE.

## Results

### CRISPR-guided ADP-ribosylation drives homologous recombination in *E.coli*

**Extended Data Fig. 1:**
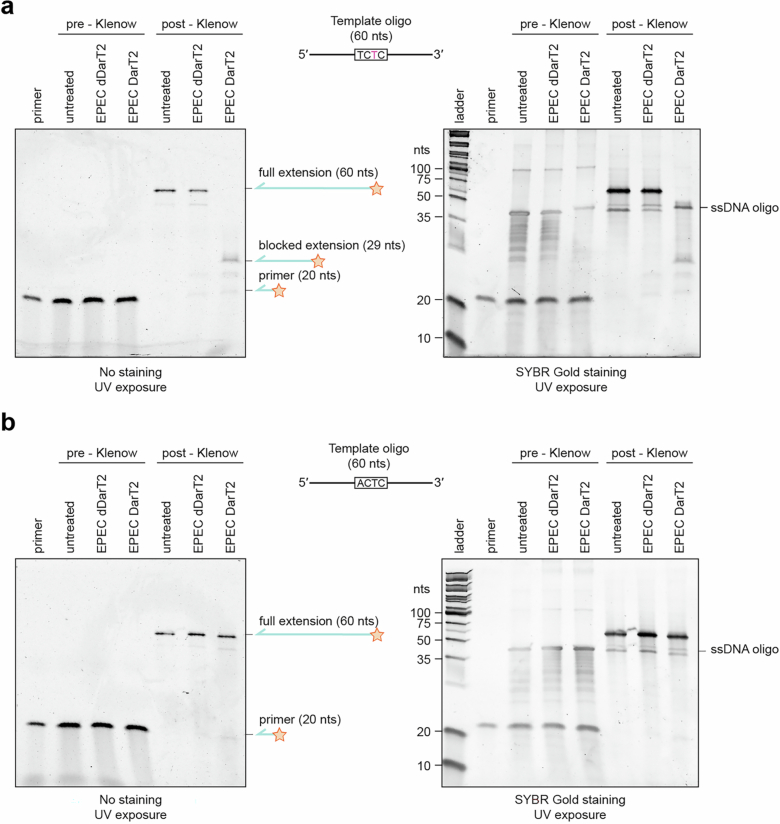
ADP-ribosylation of a 5′-TCTC-3′ motif on ssDNA by cell-free expressed EPEC DarT2 blocks DNA polymerisation by *E. coli* Klenow fragment. ssDNA oligos harboring a 5′-TCTC-3′ (**a**) or 5′-ACTC-3′ (**b**) motif were treated with wildtype or inactivated (dDarT) EPEC DarT2, annealed with a 3′-complementary Fam-tagged oligo, and treated with *E. coli* DNA Polymerase I (Klenow Fragment). Primer extension products were run on denaturing polyacrylamide gels and imaged under UV light before (left, only showing elongated Fam-tagged primer) and after (right, imaging all DNA) SYBR Gold staining. Images are representative of triplicate independent experiments. dDarT2: EPEC DarT2 with the inactivating E170A mutation. Source data

To explore the outcome of targeted DNA ADP-ribosylation, we selected the previously characterized DarT2 from enteropathogenic *E.* *coli* (EPEC) O127:H6 str. E2348/69 (ref. ^17^). EPEC DarT2 was shown to ADP-ribosylate single-stranded DNA (ssDNA) at the third position in a 5′-TYTN-3′ motif (Y = C/T), with the fourth position biased against a G^17^. Paralleling its growth-inhibitory effects in vivo, this DarT2 blocked extension by the large fragment of *E.* *coli*’s DNA polymerase I in vitro from a ssDNA template with the recognition motif (5′-TCTC-3′), whereas extension was unhindered with a mutated motif (5′-ACTC-3′) or with DarT2 containing the inactivating E170A substitution (dDarT2) (Fig. 1c,d and Extended Data Fig. 1)^17^.

To direct DNA ADP-ribosylation, we fused DarT2 to the N terminus of the protospacer-adjacent motif (PAM)-flexible (5′-NNG-3′) *Streptococcus canis* Cas9 (ScCas9) (Fig. 1e)^20^. Directing the DarT2–Cas9 fusion to a target sequence through a designed single guide (sg)RNA would localize DarT2 to the nontarget strand displaced during R-loop formation (Fig. 1e). If the non-target strand contains a 5′-TYTN-3′ motif accessible to DarT2, then the target thymine within the motif would be ADP-ribosylated and serve as a block to DNA replication. As wild-type (WT) DarT2 would arrest cell growth through genome-wide ADP-ribosylation, we included a previously reported spontaneous G49D substitution in the NAD^+^-binding loop helix (DarT2^D^) exhibiting reduced cytotoxicity^17^. To promote repair through a provided DNA template rather than the sister chromatid, we used a nickase version of Cas9 (D10A) that only cleaves the target strand and provided a plasmid-encoded repair template with ~500-bp homology arms flanking the intended edits.

As a simple readout of homologous recombination, we introduced a premature stop codon into a chromosomally integrated kanamycin resistance gene in *E.* *coli* strain MG1655 (Fig. 1f). The premature stop codon overlaps with an ScCas9 target containing the 5′-TTTC-3′ DarT2 motif and a PAM sequence, while a provided repair template with ~500-bp homology arms introduces mutations that revert the premature stop codon and remove the DarT2 motif. As part of an editing assay, plasmids encoding the editor, sgRNA and repair template are transformed into *E.* *coli* and colony counts are compared following editor induction and plating with or without kanamycin.

To set a baseline, we applied dsDNA cleavage with Cas9, which is commonly used for genome editing in bacteria^21^. As dsDNA cleavage principally removes cells that did not undergo recombination, using Cas9 resulted in an average of 76% kanamycin-resistant colonies and a 153-fold colony reduction compared to the nontargeting (NT) control (*P* = 0.0002, *n* = 3) (Fig. 1g). The nickase version of Cas9 did not deplete colony counts (3.7-fold increase relative to the NT control, *P* = 0.02, *n* = 3) but at the expense of fewer kanamycin-resistant colonies (5.1%), in line with nicking being less cytotoxic but a poor driver of homologous recombination. Binding DNA alone with a catalytically dead Cas9 (dCas9) exhibited similar colony counts to nCas9 (*P* = 0.07, *n* = 3) and did not drive any measurable editing.

**Extended Data Fig. 2:**
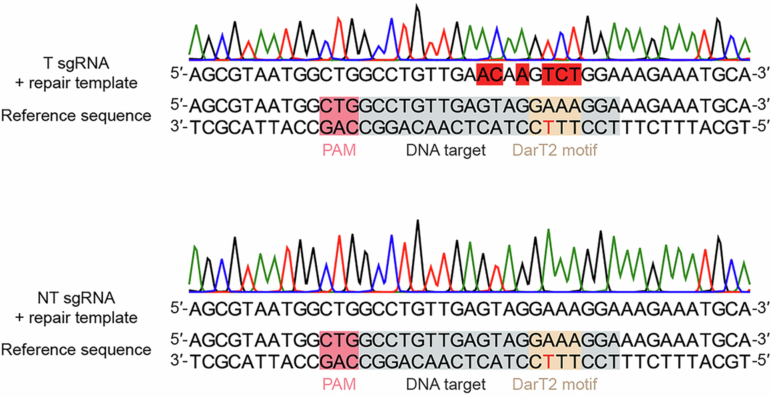
Representative Sanger sequencing results after targeting the kanR* site with DarT2^D^-nScCas9 in *E. coli* in the presence of a repair template. The target thymine in the DarT2 motif is labelled in red. The mutated bases are highlighted in red. Results are shown for a screened kanamycin-resistant colony under targeting conditions (top) and a colony under non-targeting conditions (bottom).

Turning to append editing with DarT2, the DarT2^D^–nCas9 fusion yielded an average of 97% kanamycin-resistant colonies and negligible depletion in colony counts compared to its NT control (1.7-fold increase; *P* = 0.25, *n* = 3) (Fig. 1g). Both DNA ADP-ribosylation and opposite-strand nicking were important, as conferring kanamycin resistance was less effective with nicking alone (dDarT2–nCas9, 0.18%; *P* = 0.003, *n* = 3) or ADP-ribosylation alone (DarT2^D^–dCas9, 43%; *P* = 0.029, *n* = 3) when compared to DarT2^D^–nCas9. All screened kanamycin-resistant colonies contained the intended edit (Extended Data Fig. 2). DarT2^D^ still conferred cytotoxicity, as cell counts were low even for the NT controls and increased upon deactivation of DarT2 (Fig. 1g), creating an opportunity to further attenuate the toxin. Collectively, append editing with DarT2 drives homologous recombination with a provided template in *E.* *coli*, yielding editing that outperforms traditional Cas9-based approaches but with target-independent cytotoxicity.

### Targeted ADP-ribosylation does not induce detectable base edits in *E.coli*

**Extended Data Fig. 3:**
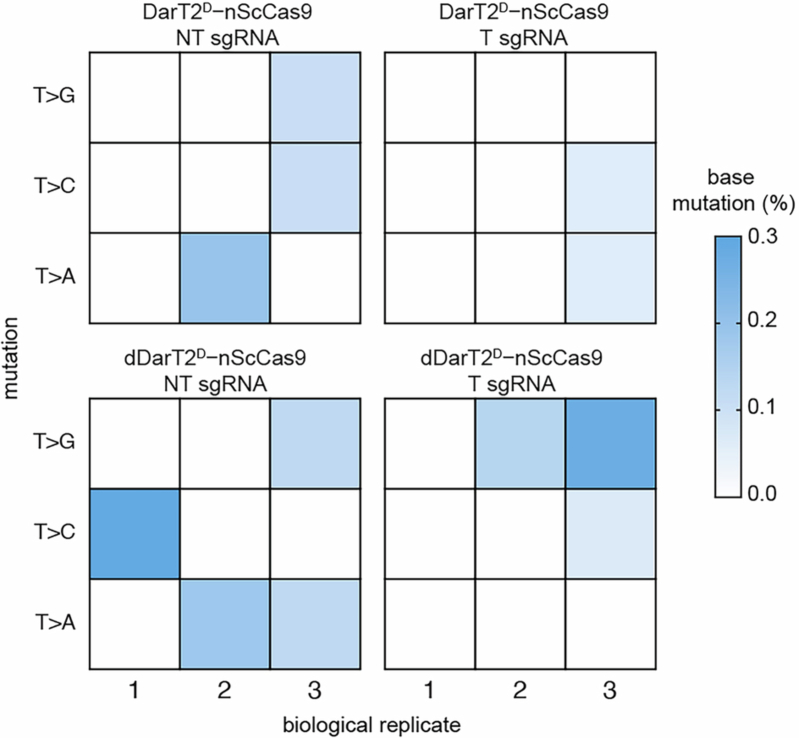
Assessing single-nucleotide variants at the ADP-ribosylated thymine targeted with the specified editor. Aligned reads are quantified that match the unedited sequence at all positions except at the ADP-ribosylated position as a percent of all aligned reads. Each biological replicate starts from a separate transformation. Source data

Our reporter assay requires homologous recombination to confer kanamycin resistance. However, chemically modifying DNA bases can lead to single-nucleotide edits as demonstrated by BEs^18,22^. We, therefore, asked whether append editing could drive editing without antibiotic selection but also induce base mutagenesis. First, we repeated the *kanR* reporter assay in the absence of kanamycin selection and performed amplicon sequencing on the target site from liquid culture (Fig. 1h). Under targeting conditions, append editing yielded 82% of total reads with the desired edit that drastically dropped with nicking alone (0.9%), paralleling the fraction of kanamycin-resistant colonies (Fig. 1g). Of the remaining reads, the few detected substitutions of the ADP-ribosylated thymine were not significantly elevated in any particular sample (*F* = 1.03, *P* = 0.39, df = 3) (Extended Data Fig. 3). As homologous recombination could overshadow base editing, we performed the assay in the absence of the repair template. However, the 16 screened colonies only yielded the original sequence (Supplementary Fig. 1). Therefore, append editing with DarT2 did not result in detectable base edits in *E.* *coli*, further supporting sole triggering of homologous recombination.

Base editing can also occur at genomic sites unrelated to the target sequence presumably through the DNA modification domain acting on temporary ssDNA^23^. Given the lack of obvious substitutions at the target site with append editing, we hypothesized that DarT2 expression would not lead to such edits associated with BEs. Culturing editor-expressing cells and performing whole-genome sequencing of three individual clones (Fig. 1i and Supplementary Table 1), a cytosine BE (CBE) yielded the expected C-to-T edits^23^, with either three or eight edits in each clone. In contrast, the ADPr-TA editor yielded no T-to-G edits and few T-to-C edits similarly to the CBE or no editor. One of the three clones with the ADPr-TA editor yielded a single T-to-A edit, whereas none were observed with the CBE or no editor. This one edit was associated with the 5′-TYTN-3′ motif, suggesting that base mutagenesis is possible but rare (Supplementary Table 1). Thus, even a highly active DarT2 that reduces cell viability (Fig. 1g) does not inherently drive base edits across the *E.* *coli* genome.

### Attenuating DarT2 alleviates cytotoxicity without compromising homologous recombination

**Fig. 2:**
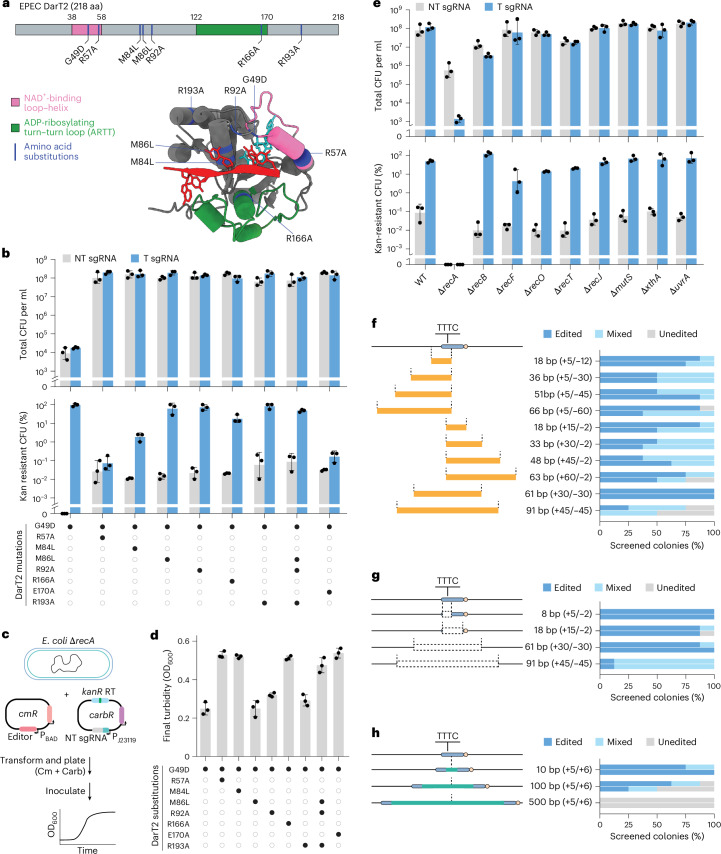
Attenuating DarT2 alleviates cytotoxicity while mediating efficient and flexible gene editing in *E.* *coli.* **a**, Predicted structure of EPEC DarT2. Tested substitutions are in blue. aa, amino acid. **b**, Impact of tested substitutions on cell viability and kanamycin resistance frequency. The experimental setup can be found in Fig. 1f. Bars and error bars represent the geometric mean ± s.d. of three independent experiments started from separate transformations. Dots represent individual measurements. **c**, Experimental setup for assessing growth defects caused by editor expression in a Δ*recA* strain of *E.* *coli*. **d**, Impact of expressing an append editor with the indicated DarT2 mutant with NT sgRNA in the Δ*recA* strain of *E.* *coli*. Endpoint OD_600_ measurements were taken after 12 h of culturing. Growth curves can be found in Supplementary Fig. 2. Bars and error bars represent the mean ± s.d. of three independent experiments started from separate transformations. Dots represent individual measurements. **e**, Impact of deleting DNA repair genes on cell viability and kanamycin resistance frequency. Bars and error bars represent the geometric mean ± s.d. of three independent experiments starting from separate transformations. Dots represent individual measurements. **f**, Introducing sequence replacements with ADPr-TA editing. **g**, Introducing deletions with ADPr-TA editing. **h**, Introducing insertions with ADPr-TA editing. **f**–**h**, Left, size and location of substitutions (orange bar), deletions (dashed box) or insertions (green bar). Numbers (for example, +5/−12) indicate the edited region in relation to the ADP-ribosylated thymine. Right, fraction of screened colonies containing the intended edit. Each bar represents one of two biological replicates starting from separate transformations, screening at least eight colonies per biological replicate. Examples of Sanger sequencing chromatograms indicating edited, mixed and unedited colonies can be found in Extended Data Fig. 4. Source data

ADPr-TAE yielded high editing efficiencies, although the expressed DarT2^D^ exhibited strong cytotoxicity (Fig. 1g). As the cytotoxicity was likely because of ADP-ribosylation of ssDNA across the genome, we aimed to attenuate DarT2 without compromising localized ADP-ribosylation and subsequent initiation of homologous recombination using structural insights and sequence conservation (Fig. 2a). While the structure of EPEC DarT2 remains to be experimentally determined, a crystal structure is available for the *Thermus* sp. 2.9 DarT2 that shares 34% amino acid identity with EPEC DarT2 (ref. ^15^). Aligning this structure with the AlphaFold-predicted structure of EPEC DarT2 (ref. ^24^), we selected a subset of residues potentially involved in binding the DNA recognition motif (M84, M86, R57, R92 and R166) or potentially flanking regions of the DNA strand not captured in the crystal structure (R193). The positively charged arginines were substituted to uncharged alanine, while the methionines were substituted to leucine to disrupt the coordinating sulfur while preserving the residue’s hydrophobicity and chain length. Testing these substitutions in combination with G49D as part of the kanamycin resistance reversion assay (Fig. 1f), we found that all improved cell viability (Fig. 2b). At the same time, three of the substitutions (M86L, R92A and R193A) maintained the fraction of kanamycin-resistant colonies comparable to the original G49D (*P* = 0.77, 0.51 and 0.27, respectively, *n* = 3) (Fig. 2b), representing candidates for further use with append editing.

Viability was greatly enhanced across the single-substitution variants, yet DarT2 may still exert target-independent ADP-ribosylation that could have more subtle effects on cell growth and behavior. We, therefore, generated cells hypersensitive to ADP-ribosylation by deleting the core repair gene *recA* to disable homologous recombination and assessed cell growth when expressing each ADPr-TAE variant under non-targeting conditions (Fig. 2c and Supplementary Fig. 2). While growth rates in the exponential phase were similar (Supplementary Fig. 2), we observed marked differences upon entry into the stationary phase. In particular, amino acid substitutions that previously compromised editing (M84L, R57A and R166A) yielded final turbidities paralleling the inactivating E170A (*P* = 0.35, 0.65 and 0.22, respectively, *n* = 3) (Fig. 2d and Supplementary Fig. 2). In contrast, substitutions that previously showed high editing efficiencies (M86L, R92A and R193A) exhibited a final turbidity similar to G49D alone (*P* = 0.99, 0.05 and 0.17, respectively, *n* = 3) and lower than E170A. We, therefore, combined the high-editing-efficiency substitutions (M86L, R92A and R193A) into a four-substitution version of DarT2, DarT2^DLAA^. This version maintained cell viability and a high frequency of kanamycin-resistant colonies (49%) in *E.* *coli* MG1655 (Fig. 2b). Moreover, in the *recA*-deletion (Δ*recA*) strain, the append editor with DarT2^DLAA^ restored final turbidity to approach that of the editor lacking ADP-ribosylation (E170A; *P* = 0.09, *n* = 3) (Fig. 2d).

By improving cell viability and growth in a strain in which homologous recombination was fully disabled, the append editor with DarT2^DLAA^ afforded the opportunity to probe the genetic basis of templated-mediated editing. Prior work on the cytotoxicity of DarT2^D^ in *E.* *coli* revealed a key role by RecF and possibly nucleotide excision repair^17^. However, the involved DNA repair pathways as part of targeted ADP-ribosylation with opposite-strand nicking could differ. Within the kanamycin reversion assay (Fig. 1f), *recA* was essential for editing and even showed some reduction in colony counts under non-targeting conditions (Fig. 2e). Disrupting the RecBCD branch of recombination (Δ*recB*) reduced viability but also increased the frequency of kanamycin-resistant colonies, suggesting a role in survival in the absence of recombination with the provided repair template. In contrast, disrupting the alternative RecFOR recombination pathway (Δ*recF* and Δ*recO*) reduced editing relative to the WT (one-sided Welch’s *t*-test, *P* = 0.048 and 0.001, respectively, *n* = 3) but not viability for *recF* (one-sided Welch’s *t*-test, *P* = 0.40, *n* = 3), suggesting involvement in templated recombination. Disrupting RecA-independent RecT recombination (Δ*recT*) significantly reduced both viability and editing (one-sided Welch’s *t*-test, *P* = 0.002 and 0.003, respectively, *n* = 3), suggesting involvement in both survival and templated recombination. Lastly, disruption of the DNA repair exonuclease RecJ (Δ*recJ*), mismatch repair (Δ*mutS*), base excision repair (Δ*xthA*) and nucleotide excision repair (Δ*uvrA*) did not impact editing (one-sided Welch’s *t*-test, *P* = 0.89, 0.68 and 0.81, respectively, *n* = 3) or viability (one-sided Welch’s *t*-test, *P* = 0.87, 0.24 and 0.93, respectively, *n* = 3) relative to the WT. These findings implicate multiple recombination pathways as part of ADPr-TAE in *E.* *coli*.

### Attenuated ADP-ribosylation enables flexible and noncytotoxic genome editing in bacteria

Append editing with DarT2^DLAA^ efficiently reverted the premature stop codon in the kanamycin reversion assay. However, the reliance on homologous recombination lends to a much broader range of edits in different genes and bacteria. We, therefore, explored the bounds of ADPr-TA editing. For simplicity, editing was performed around the premature stop codon in the kanamycin reversion assay. When testing edits beyond reversion of the stop codon, editing efficiency was determined without kanamycin selection by assessing the size of the target site or sequence of individual colonies.

**Extended Data Fig. 4:**
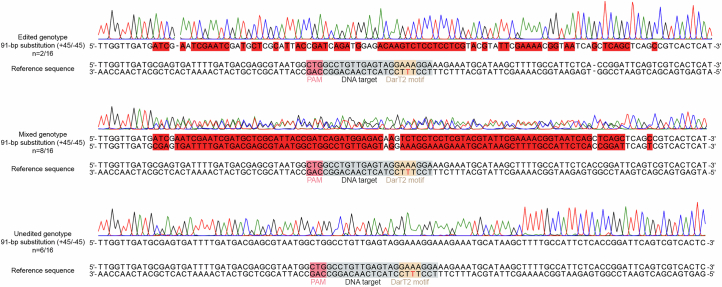
Examples of edited, mixed, and unedited genotypes for sequence replacement with DarT2^DLAA^-nScCas9 targeting kanR* in *E. coli.* Representative Sanger sequencing results from *E. coli* colonies transformed with a plasmid that contained a repair template to mediate the replacement of 91 bp at the kanR* site; 45 bp upstream and 45 bp downstream (+45/-45) of the ADP-ribosylated thymine.

**Extended Data Fig. 5:**
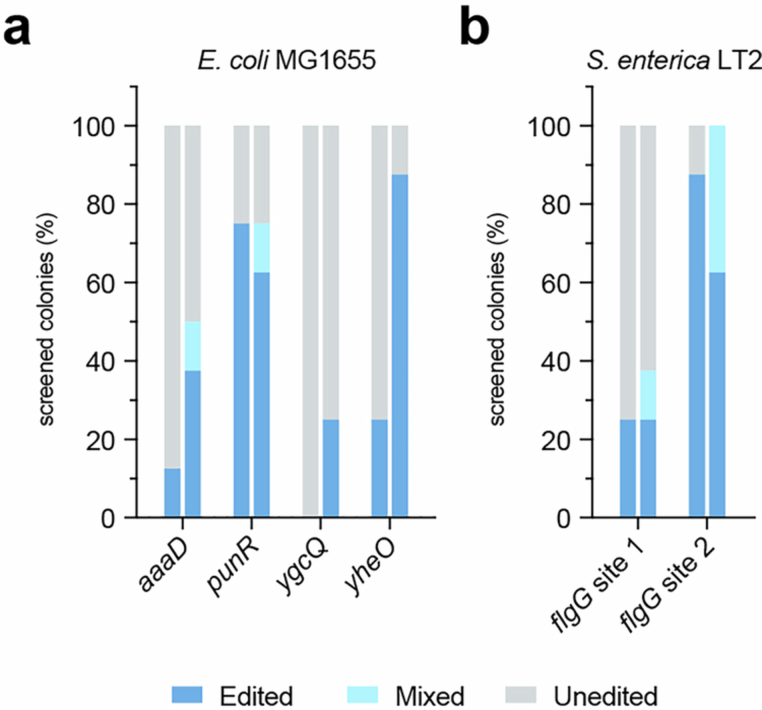
ADPr-TAE with DarT2^DLAA^-nScCas9 at different sites in *E. coli* MG1655 and at two sites in the *flgG* gene in *S. enterica* LT2. **a**, ADPr-TA editing through substitution at the *aaaD*, *punR*, *ygcQ*, and *yheO* genes of *E. coli* MG1655. All substitutions introduced silent mutations. **b**, ADPr-TA editing through substitution (*flgG* site 1) or deletion (*flgG* site 2) at the *flgG* gene of *S. enterica* LT2. All experiments for *E. coli* and *S. enterica* were performed in duplicate, and eight colonies were screened through colony PCR and Sanger sequencing per duplicate experiment.

Beginning with the homology arms, condensing their length from ~500 to 100 bp reduced the frequency of kanamycin resistance from 86% to 28%, whereas arm lengths of 50 bp and below exhibited virtually no kanamycin resistance (Supplementary Fig. 3). Continuing with ~500-bp homology arms, we tested increasingly larger replacements, deletions and insertions (Fig. 2f–h). Replacements extending up to 60 bp upstream or downstream of the target site or 91 bp spanning the target site were present in 80–100% and 50–75% of screened colonies, respectively, either as complete or partial conversions (Fig. 2g and Extended Data Fig. 4). Separately, deletions up to 91 bp were present in 90–100% of screened colonies, albeit with a high fraction of partial conversion with the largest deletion. Lastly, insertions of 10 bp and 100 bp were present in 100% and 50–90% of screened colonies, respectively. No colonies contained an insertion of 500 bp (Supplementary Fig. 4), indicating an upper limit to recombination. Editing was not limited to this target site in *E.* *coli*, as we could introduce substitutions at four additional targeted genes in *E.* *coli* (Extended Data Fig. 5a) and one targeted gene in the pathogen *Salmonella enterica* (Extended Data Fig. 5b). Collectively, ADPr-TAE can introduce ranging replacements, insertions and deletions in bacteria without sacrificing viability.

### Targeted ADP-ribosylation preferentially drives base mutagenesis in yeast and plants

**Fig. 3:**
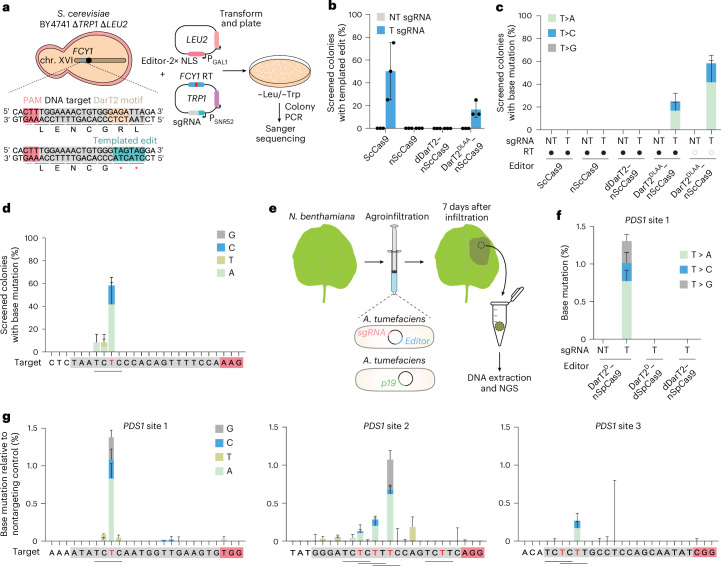
Programmable DNA ADP-ribosylation primarily drives base mutagenesis in yeast and plants. **a**, Experimental setup for introducing a six-base replacement with two adjacent premature stop codons in the *FCY1* gene of *S.* *cerevisiae*. NLS, nuclear localization signal. **b**, Impact of ADPr-TAE on templated recombination in the presence of an RT. Bars and error bars represent the mean and s.d. of three independent experiments started from separate transformations. Dots represent individual measurements. **c**, Impact of ADPr-TAE on mutagenesis of the ADP-ribosylated thymine in the presence or absence of an RT. **d**, Frequency of base mutations across the sgRNA target. Each black bar specifies DarT2 recognition motifs, while the red base specifies the ADP-ribosylated base within the motif. Representative Sanger sequencing chromatograms can be found in Extended Data Fig. 6a,b. In **c** and **d**, bars and error bars represent the mean and s.d. of three independent experiments started from separate transformations. **e**, Experimental setup for assessing ADPr-TAE without an RT in *N.* *benthamiana*. **f**, Frequency of base mutagenesis of the ADP-ribosylated thymine in the sgRNA1 target in the *PDS1* gene compared to the NT control. **g**, Frequency of base mutations across the DNA target for sgRNA1–sgRNA3 compared to the NT control. The location of base mutations under targeting and non-targeting conditions can be found in Supplementary Fig. 6. In **f** and **g**, bars and error bars represent the mean and s.e.m. of three independent experiments started from separate transformations. Source data

**Extended Data Fig. 6:**
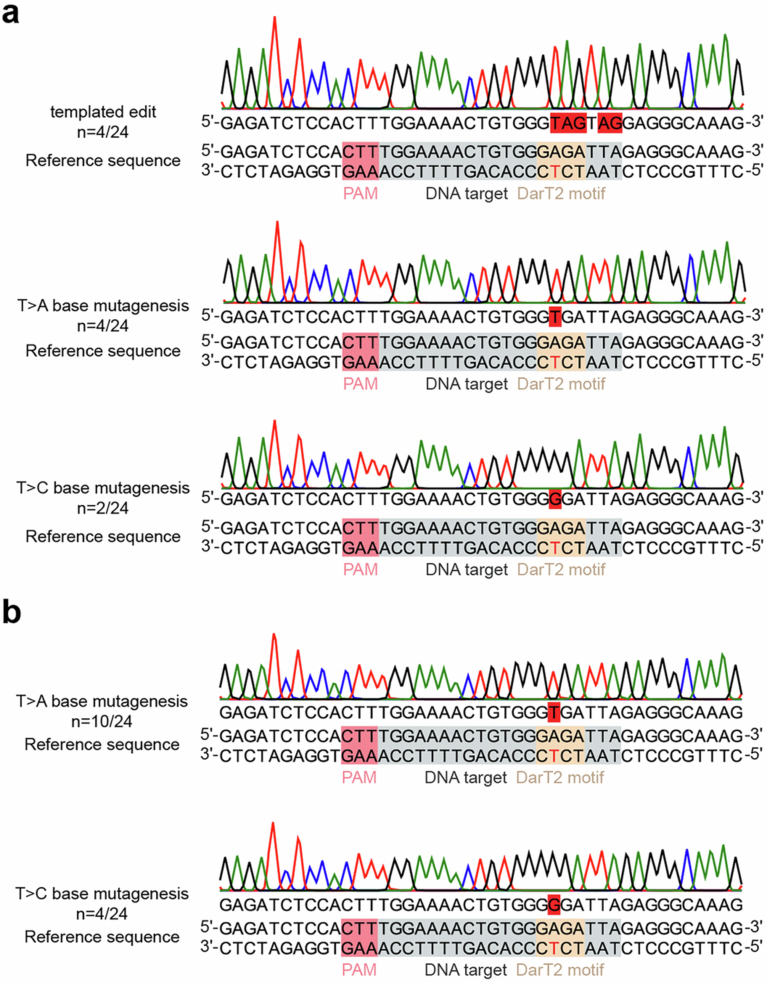
Representative genotypes after targeting the *FCY1* gene with DarT2^DLAA^-nScCas9 in *S. cerevisiae* in the presence or absence of a repair template. **a**, Editing in the presence of the repair template. **b**, Editing in the absence of the repair template. For each condition, 24 colonies were screened by Sanger sequencing of the target site, and the frequency of each specified edit is shown.

Given that append editing drove templated recombination in bacteria, we asked whether eukaryotes would undergo similar editing outcomes. Beginning with the baker’s yeast *Saccharomyces cerevisiae* cultured as a haploid, we transformed plasmids encoding the DarT2^DLAA^ append editor, an sgRNA and a repair template with ~250-bp homology arms to introduce a premature stop codon as part of six substitutions in the *FCY1* gene. Individual colonies were then screened on the basis of Sanger sequencing of the target site (Fig. 3a and Extended Data Fig. 6a,b). Append editing with DarT2^DLAA^–nScCas9 yielded templated edits in only 17% of the screened colonies, a reduced frequency compared to 50% generated via dsDNA breaks with ScCas9 (Fig. 3b). No edited colonies were obtained under non-targeting conditions or with DNA nicking alone, affirming the necessity of either dsDNA breaks or targeted ADP-ribosylation for templated editing.

Beyond templated edits achieved with targeted ADP-ribosylation, we also observed a distinct set of edits in 25% of the screened colonies: conversion of the ADP-ribosylated thymine into a different base (Fig. 3c and Extended Data Fig. 6a). These base substitutions principally occurred at the thymine expected to undergo ADP-ribosylation by DarT2, with the modified base becoming an A (67%) or a C (33%) (Fig. 3c). Such edits were absent with any of the other tested editors (Fig. 3c). Homologous recombination and base mutagenesis represented mutually exclusive repair outcomes, as removing the repair template enhanced the mutagenesis frequency without altering the location and distribution of mutations (Fig. 3c,d and Extended Data Fig. 6b). Base mutation was also observed when targeting sites within the genes *ALP1* and *JSN1*, albeit at lower frequencies (Supplementary Fig. 5a,b). Thus, in yeast, append editing drives either homology-directed repair (HDR) or mutagenesis of the ADP-ribosylated thymine.

The outcomes of append editing in yeast represented a major deviation from what we observed in tested bacteria and could reflect distinct editing outcomes in eukaryotes at large. However, in contrast to higher eukaryotes, *S.* *cerevisiae* engages in nonhomologous end joining less frequently and lacks poly(ADPr) polymerases involved in dsDNA break repair that add and extend ADPr groups on DNA ends^25,26^. We, therefore, assessed the impact of ADPr-TAE in the model plant *Nicotiana benthamiana*. As a simple and fast assay, *Agrobacterium* constructs encoding the append editor were injected into *N.* *benthamiana* leaves, after which the type and frequency of edits were assessed by targeted amplicon sequencing of transfected tissues (Fig. 3e). In this setup, no repair template was included given the generally low frequencies of homologous recombination in this type of transfection assay in plants^27^. Additionally, the ScCas9 component of the append editor was exchanged for *Streptococcus pyogenes* Cas9 (SpCas9) to use available constructs.

Despite expectedly low transfection efficiencies, we could measure substitution of the ADP-ribosylated thymine as the dominant outcome in 1.4% of reads targeting the *PDS1* gene (Fig. 3f,g). This thymine was converted to the three other bases but with a bias toward A (59%) over C (19%) and G (22%). Testing two other target sites within *PDS1*, including one containing multiple DarT2 motifs, resulted in similar mutagenesis of the ADP-ribosylated T, with a bias toward A (Fig. 3g and Supplementary Fig. 6). Indels were observed under targeting conditions but at frequencies 6–80-fold lower than base mutagenesis (Supplementary Fig. 7). Thus, append editing can drive mutagenesis of the ADP-ribosylated base in both yeast and plants, reflecting distinct editing outcomes from those we observed in bacteria.

### Targeted ADP-ribosylation drives base mutagenesis in human cells lacking TARG1

**Fig. 4:**
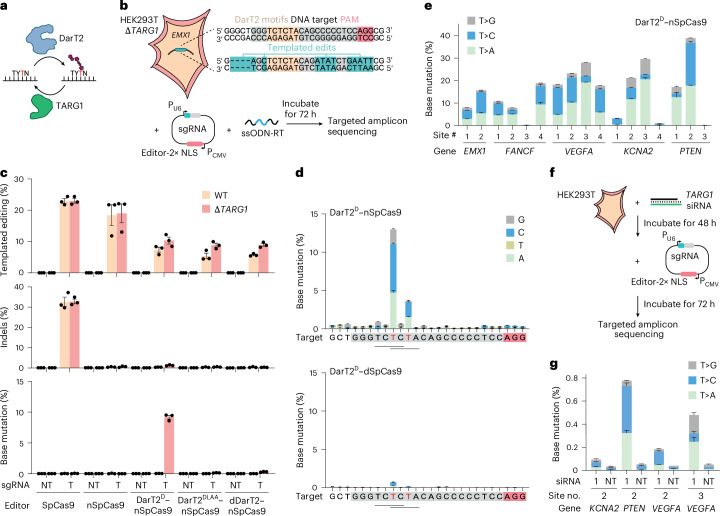
Programmable DNA ADP-ribosylation preferentially drives base mutagenesis in human cells lacking TARG1. **a**, Reversion of ADP-ribosylation of ssDNA in human cells by the TARG1 protein. **b**, Experimental setup for introducing edits in the *EMX1* gene in HEK293T cells using an oligonucleotide RT. ssODN, single-stranded oligodeoxynucleotide. **c**, Extent of templated recombination (top), indel formation (middle) or base mutagenesis (bottom) using *EMX1* sgRNA1 in HEK293T cells with TARG1 intact (WT) or disrupted (Δ*TARG1*). Bars and error bars represent the mean and s.e.m. of three independent transient transfections without selection or sorting. **d**, Frequency of base substitutions across the sgRNA target in the absence of the oligonucleotide RT. Results are shown with DNA nicking by Cas9 intact (top) or disabled (bottom). **e**, Extent of base mutagenesis of the ADP-ribosylated thymine across 17 target sites in five genes. **f**, Experimental setup for editing in HEK293T cells using siRNAs to reduce *TARG1* levels. **g**, Extent of base substitutions in HEK293T cells following siRNA-mediated silencing of *TARG1* expression. In **d**, **e** and **g**, bars and error bars represent the mean and s.e.m. of three independent transient transfections without selection or sorting. Source data

As a final but important branch of eukaryotes, we sought to explore append editing in human cells. Unlike *S. cerevisiae* and *N. benthamiana*, human cells possess an *o*-acyl-ADPr deacylase (OARD1), also known as TARG1, that was previously shown to reversibly remove the ADPr moiety appended to thymines by DarT2 (Fig. 4a)^28^. We, therefore, began by assessing ADPr-TAE in human cells with an intact or disrupted *TARG1* gene (Supplementary Fig. 8) using SpCas9 on the basis of available constructs. Plasmid constructs encoding an SpCas9-based editor and an sgRNA were transiently transfected into HEK293T cells and editing was assessed through next-generation sequencing of the target site in *EMX1* without sorting or selection of transfected cells (Fig. 4b). An oligonucleotide repair template specifying a nine-base substitution and four-base deletion was included to evaluate both homologous recombination and base mutagenesis in parallel.

Using SpCas9 in HEK293T cells as a baseline, we observed matching extents of templated edits (22%) and small indels (32%), with no significant difference in the absence of TARG1 (*P* = 0.99 and 0.94, respectively, *n* = 3) (Fig. 4c). Nicking similarly generated a high level of templated edits whether or not TARG1 was intact (18%) but with minimal small indels (0.4%) because of the lack of dsDNA breaks. The append editor with DarT2^D^ also yielded templated edits, with the editing frequency increasing from 7% to 10% by disrupting TARG1. However, no significant differences were observed for append editors with the attenuated DarT2^DLAA^ or with dDarT2 (*P* = 0.32 and 0.33, respectively, *n* = 3), suggesting that the templated edits were driven primarily through DNA nicking rather than DNA ADP-ribosylation.

At the same time, the ADPr-TA editor with DarT2^D^ yielded 9% base substitutions specifically at the ADP-ribosylated thymine within two overlapping DarT2 recognition motifs, but only with TARG1 disrupted (Fig. 4c). Base substitutions were negligible with DarT2^DLAA^ (0.2%) or dDarT2 (0.3%), suggesting that higher levels of ADP-ribosylation were necessary to drive editing (Fig. 4c). Indel frequencies for ADPr-TAE were slightly elevated over nCas9 with TARG1 disrupted (1.5% versus 0.9%; *P* = 0.03, *n* = 3) but still 22-fold lower than that observed with Cas9 (33%) (Fig. 4c), indicating that the principal repair outcome of ADP-ribosylation and opposite-strand nicking is base mutagenesis. Thus, ADPr-TAE in HEK293T cells drives base mutagenesis similarly to what is observed in plants and yeast, but only in the absence of TARG1.

**Extended Data Fig. 7:**
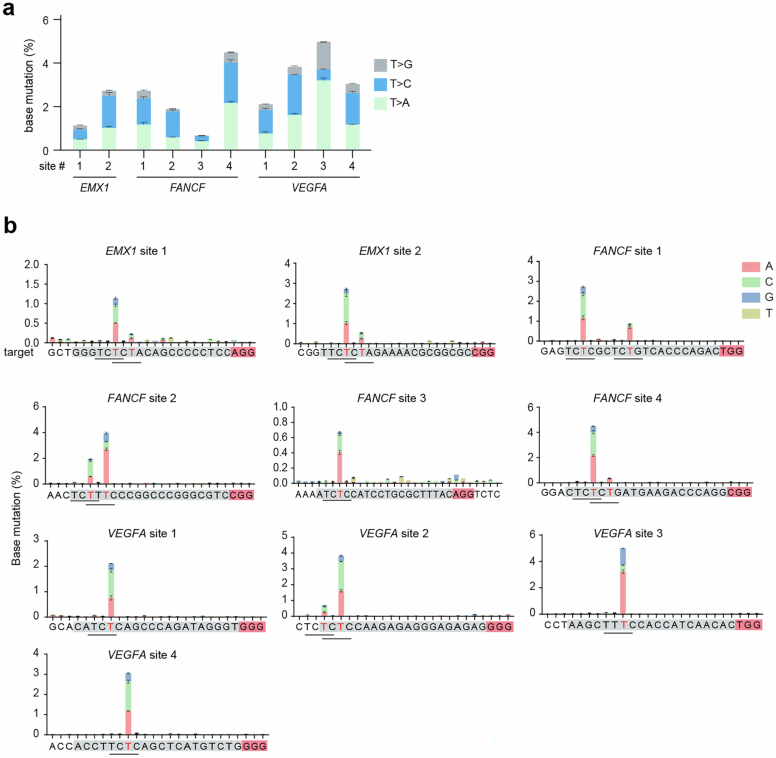
Base substitution frequencies at the ADP-ribosylated thymine with DarT2^D^-nSpCas9 across different targets in *EMX1*, *FANCF* and *VEGFA* genes in U2OS Δ*TARG1* cells. **a**, Frequencies of base substitutions at the ADP-ribosylated thymine. **b**, Locations of base substitutions across the target site. Horizontal bars indicate DarT2 recognition motifs, with ADP-ribosylation of the thymine at the third position. Bars and error bars in a and b represent the mean and s.e.m. of three independent replicates without selection or sorting. Source data

**Extended Data Fig. 8:**
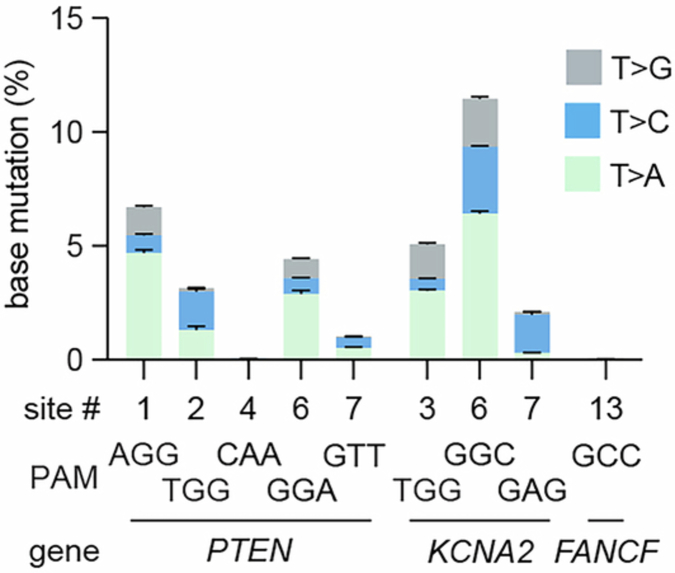
Append editing using DarT2^D^ fused with the nearly PAM-less nSpRYCas9. Frequencies of base substitutions at the ADP-ribosylated thymine are shown along with the PAM. Bars and error bars represent the mean and s.e.m. of three independent replicates without selection or sorting. Source data

As different oligonucleotide templates revealed reduced templated repair with increased base mutagenesis (Supplementary Fig. 9), we repeated the editing assay without the oligonucleotide template. Base mutagenesis at both modified thymines increased to 16% (Fig. 4d), with conversion to either A or C at similar frequencies. Additionally, base mutagenesis was reduced 20-fold to 0.8% in the absence of DNA nicking, indicating the importance of the nick (Fig. 4d). We also observed a low frequency of deletions up to ~25 bp that were elevated with DNA nicking (Supplementary Fig. 10), paralleling observations with BEs^29^. Probing base mutagenesis beyond this target site, we performed transient transfections without the oligonucleotide template at 16 additional target sites in five genes containing one or more DarT2 recognition motifs (Fig. 4e and Supplementary Fig. 11). We observed measurable editing at all but two of these sites, with editing frequencies reaching up to 39% (Fig. 4e and Supplementary Fig. 11). Similar trends were observed in U2OS ∆*TARG1* cells^28^, with generally lower editing frequencies (up to 5.0%) likely because of lower transfection efficiencies (Extended Data Fig. 7a,b). We could also couple DarT2 with the nearly PAM-less SpRY variant of SpCas9 (ref. ^30^) to drive base substitutions through non-NGG PAMs (Extended Data Fig. 8).

Given the need to delete *TARG1* to observe editing, we assessed the ability to transiently silence TARG1 expression to promote editing in WT HEK293T cells using RNA interference (Fig. 4f). Of three tested small interfering RNAs (siRNAs) that each reduced *TARG1* transcripts by at least 75% (Supplementary Fig. 12a), one siRNA yielded significant editing across all four tested target sites (Fig. 4g and Supplementary Fig. 12b). Editing was greatly diminished compared to that in cells lacking *TARG1* (for example, 39% in HEK293T ∆*TARG1* versus 0.8% in WT cells with TARG1-siRNA at *PTEN* site 2), suggesting that residual TARG1 blocks editing with DarT2. Overall, these results show that append editing with DarT2 principally drives substitution of the ADP-ribosylated base in human cells, with TARG1 posing a barrier to editing.

### Targeted ADP-ribosylation mediates flexible, distinct and specific editing in mammalian cells

**Fig. 5:**
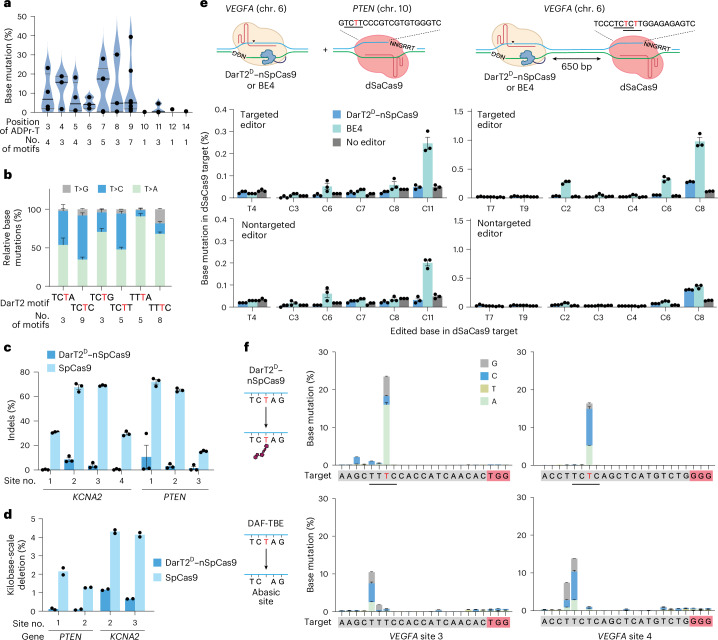
Programmable ADP-ribosylation enables specific and differentiated editing in human cells lacking TARG1. All experiments were conducted in HEK293T Δ*TARG1* cells. **a**, Extent of base mutagenesis based on the relative location of ADPr-T according to **e**. Cumulative thymine base editing across 21 sgRNA targets, within 37 5′-TYTN-3′ motifs at positions 3–14 (with position 1 is at the PAM-distal end). Solid black lines represent the median and gray lines represent the quartiles. Each dot represents the mean of three independent transient transfections without selection or sorting for a given sgRNA. **b**, Relationship between the outcome of base mutagenesis and the DarT2 recognition sequence according to **e**. Distributions were calculated for base mutations occurring at 33 DarT2 recognition motifs across 21 sgRNAs. **c**, Frequency of indels for DarT2^D^–nSpCas9 compared to SpCas9 at the same target sites. **d**, Frequency of kilobase-scale deletions for DarT2^D^–nSpCas9 compared to SpCas9 at the same target sites. **e**, Guide-independent base substitutions at editable bases within orthogonal R-loops formed with dSaCas9 distant from (left) or close to (right) the target site. Editing was assessed through append editing (DarT2^D^–nSpCas9) or cytosine base editing (BE4) in HEK293T Δ*TARG1* cells. Editing was significantly higher at position C11 of the R-loop in *PTEN* with the targeted BE4 (*P* = 0.016) or a nontargeted BE4 (*P* = 0.003) and at positions C2 (*P* = 0.003), C6 (*P* = 0.007) and C8 (*P* = 0.006) of the R-loop in *VEGFA* for the targeted BE4. **f**, Frequency of base substitutions using programmable ADP-ribosylation (DarT2^D^–nSpCas9) or glycosylation (DAF-TBE) of thymine in HEK293T Δ*TARG1* cells. Bars and error bars in **b**, **c**, **e** and **f** represent the mean and s.e.m. of three independent transient transfections without selection or sorting. Bars in **d** represent the mean of the two independent transient transfections without selection or sorting. Source data

**Extended Data Fig. 9:**
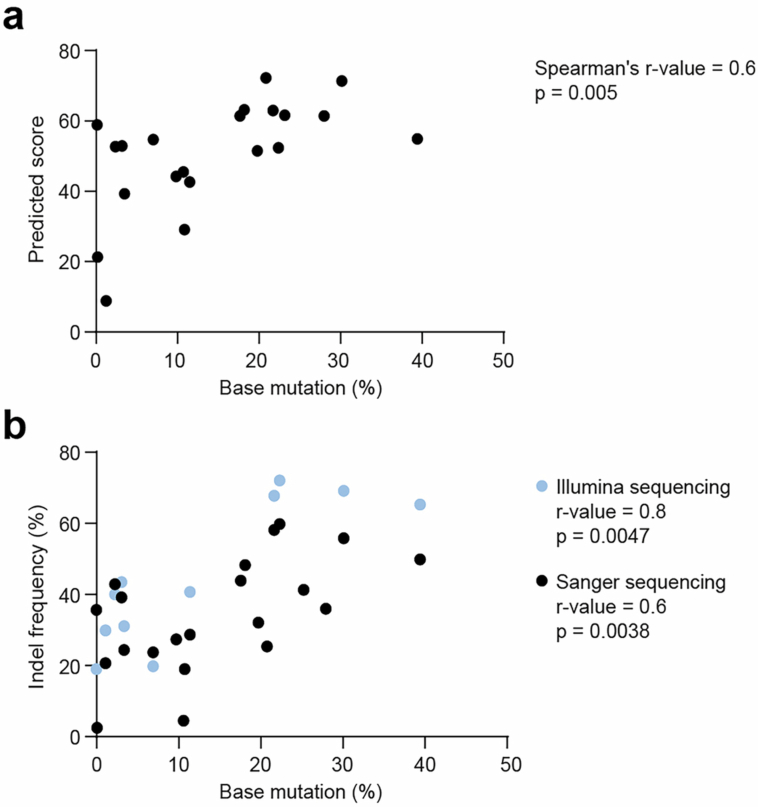
Correlation between measured or predicted indel formation at target sites with SpCas9 and ADPr-TAE-mediated base mutation. **a**, Correlation between ADPr-TAE-mediated base mutation frequency and on-targeting scores predicted for indel formation with traditional Cas9. Each dot represents the mean of base mutation frequencies (n = 3) observed experimentally, plotted against scores predicted through the Rule Set 2 scoring method^2^, for a specific genomic target. **b**, Correlation between ADPr-TAE base mutation frequency and indels generated by SpCas9. Each dot represents the mean of base mutation frequencies (n = 3), determined by Illumina sequencing against Indels determined through Illumina and Sanger sequencing methods (n = 3), for a specific genomic target. Source data

The expanded set of target sites allowed us to explore unique features of base mutagenesis. Across these sites, editing principally occurred at the modified thymine falling between positions 3 and 9 of sgRNA guide (Fig. 5a). For targets with multiple DarT2 recognition motifs, co-occurring mutations were observed 1.1-fold to 5.1-fold more frequently than expected if the motifs could be edited independently (Supplementary Fig. 13). Across these sites, we noticed distinct mutagenesis distributions that strongly depended on the DarT2 recognition motif (Fig. 5b). Specifically, 5′-TCTN-3′ motifs were associated with similar conversion frequencies to A and C. In contrast, 5′-TTTN-3′ were associated with a strong bias toward A, with secondary edits biased toward C (5′-TTTA-3′) or equally split between C and G (5′-TTTC-3′). We further assessed the frequency of small indels at selected target sites. Compared to Cas9, the append editor resulted in 6–110-fold lower indel frequencies (Fig. 5c). Indel frequencies measured by next-generation sequencing or predicted using the Rule Set 2 scoring method^31^ at each target site with Cas9 correlated with base mutagenesis frequencies (Spearman correlation, *ρ* = 0.80 and 0.58, respectively) (Extended Data Fig. 9), indicating that indel formation with Cas9 offers a starting point to identify efficient sites for append editing. Additionally, the append editor resulted in 3–21-fold fewer kilobase-scale deletions compared to Cas9, as detected through long-read sequencing (Fig. 5d and Supplementary Fig. 14)^32^.

**Extended Data Fig. 10:**
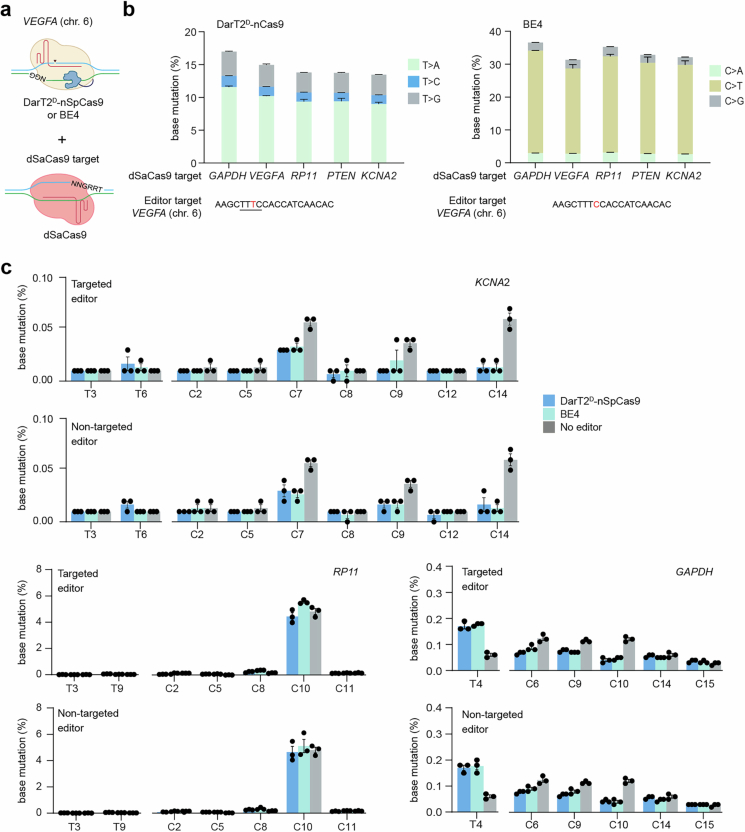
Assessing sgRNA-independent off-target editing via DNA ADP-ribosylation. **a**, General configuration for assessing sgRNA-independent off-targeting through the generation of an orthogonal R-loop via dSaCas9. **b**, On-target editing activities under targeting conditions. **c**, Base mutation across dSaCas9 target sites under targeting and non-targeting conditions by the editor. No editor: expression of the targeting sgRNA without an editor. Bars and error bars in b and c represent the mean and s.e.m. of three independent replicates without selection or sorting. Source data

Beyond unintended on-target edits, we also investigated guide-independent off-target effects knowing that errant DNA ADP-ribosylation could drive base substitution (Fig. 5e). As a point of comparison, we used the CBE BE4 previously demonstrated to introduce cytosine base edits at orthogonal R-loops^23^. Creating orthogonal R-loops at each of five sites using the dead Cas9 from *Staphylococcus aureus* (dSaCas9), BE4 generated cytosine base substitutions significantly more often than a no-editor control at two of the sites (Fig. 5e and Extended Data Fig. 10). Intriguingly, base substitutions in the R-loop were highest when BE4 was targeted ~650 bp upstream of the R-loop (Fig. 5e and Extended Data Fig. 10), suggesting enhanced frequencies of sgRNA-independent editing in the vicinity of the target site. In contrast, the append editor did not result in any increase in base mutations at the thymine within the DarT2 motif across all five sites compared to the no-editor control. These results are in line with the need for opposite-strand nicking to drive append editing at the target site (Fig. 4d).

BEs using thymine glycosylases were recently reported^33–35^, raising the question how editing of the modified thymine compares between base excision and ADP-ribosylation. We, therefore, assessed editing outcomes at two target sites in HEK293T ∆*TARG1* cells with our append editor and the deaminase-free (DAF) thymine BE (TBE) as a representative example (Fig. 5f)^33^. DAF-TBE edited multiple thymines within the target, with the most efficient editing at position 5 of the sgRNA guide. The append editor edited only the thymine in the recognized motif, with higher editing than any single thymine with DAF-TBE at both sites (*P* = 0.0001 and 0.052). Interestingly, the editing profiles were distinct, with the DAF-TBE predominantly yielding T-to-C or T-to-S edits^33^ compared to predominantly T-to-A or T-to-M edits using append editing. Both editors exhibited similarly low levels of indel formation and large deletions (Supplementary Figs. 15 and 16). These results show that ADP-ribosylation and base excision of thymine drive distinct editing outcomes.

## Discussion

**Fig. 6:**
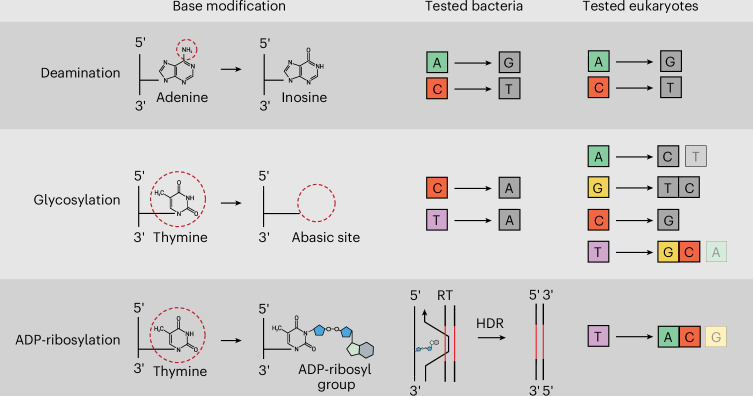
Programmable ADP-ribosylation of thymine generates distinct editing outcomes in bacteria and eukaryotes compared to deaminase and glycosylase base editing. Editors with deaminases include ABEs^60^ and CBEs^61^, while editors with glycosylases include A-to-Y BEs^41^, glycosylase BEs^62^, adenine transversion BEs^63^, glycosylase-based guanine BEs^64^, glycosylase-based TBEs, glycosylase-based CBEs^35^, DAF-TBEs, DAF-CBEs^33^, thymine DNA glycosylase-based editor, cytosine DNA glycosylase-based editor^42^ and TBEs^34^. Nucleotides representing edits are colored to help compare the glycosylation and ADP-ribosylation of thymine.

In this work, we explored the impact of appending chemical moieties to target DNA as a distinct yet broad approach for precision editing, what we call append editing. As a first example, we used the bacterial toxin DarT2 to mediate ADPr-TAE. When paired with opposite-strand nicking, ADPr-TAE introduced precise edits through homologous recombination in tested bacteria, allowing the creation of templated edits (Fig. 6). While this strategy also drove templated recombination in yeast, the predominant outcome was mutagenesis of the ADP-ribosylated thymine. Base mutagenesis was similarly observed in plants and mammalian cells, with a general bias toward substitution to A or C (Fig. 6). Although the exact underlying repair pathways in eukaryotes remain to be identified (for example, nucleotide excision repair or translesion synthesis), homologous recombination can at least be excluded. This divergence in repair pathways contrasts with other genome-editing approaches that engage equivalent repair pathways across organisms and result in similar types of edits, supporting append editing as a distinct entry in the genome-editing toolbox.

Furthermore, ADPr-TAE offers unique opportunities for genome editing in bacteria (Fig. 6) exemplified by the broad range of generated sequence replacements, deletions and insertions. This form of editing did not sacrifice colony counts compared to traditional dsDNA cleavage^36^, offered broader edits without perturbing DNA repair compared to prime editing^37,38^ and omitted fixed scars compared to CRISPR-associated transposons^39^. Given these distinctions, ADPr-TAE is well suited for generating large chromosomal libraries and multiplexed editing or multibase editing in nonmodel bacteria^40^.

In yeast, plant and human cells, ADPr-TAE operates closest to BEs yet offers distinct editing avenues. BEs to date rely on base deaminases or glycosylases that convert T (or A on the opposite strand) into C (adenosine deaminase)^18^, G (adenine glycosylase)^41^ or C/G (thymine glycosylase), with extensive bystander editing^33–35,42^. In contrast, ADPr-TAE converts T to A or A/C depending on the organism and sequence context, with minimal bystander edits. T-to-A editing is particularly unique, where ADPr-TAE could potentially revert 789 of the verified pathogenic single-nucleotide variants (SNVs) across 355 genes in the ClinVar database^43^ otherwise off-limits through existing TBEs. While the current DarT2 recognition motif would capture a fraction of these SNVs (that is, 30 T-to-A and 447 T-to-C mutations) (Supplementary Table 3), relaxing the motif through ortholog mining or protein engineering could access a greater set. A stringent motif can also be beneficial, such as when reversing pathogenic mutations susceptible to bystander edits. In particular, ADPr-TAE could create a single desired T-to-C edit in a stretch of three thymines (for example, pathogenic mutation in the third T of 5′-TTTG-3′ (c.103C>T, c.4396C>T, c.4852C>T, c.5188C>T, c.5623C>T, c.742C>T and c.748C>T) or 5′-TTTA-3′ (c.1537C>T, c.3346C>T, c.3673C>T, c.3826C>T, c.4603C>T, c.5473C>T and c.5599C>T) in the *ATM* gene underlying ataxia telangiectasia^44^), while current adenine BEs (ABEs) would generate unwanted edits across the thymines. TARG1 poses an immediate barrier to ADPr-TAE in human cells; while we marginally circumvented TARG1 using transient gene silencing with RNA interference^45^, more potent approaches such as the development of specific peptide or chemical inhibitors^46^ or dominant-negative mutants such as those used to inhibit mismatch repair^47^ may be required.

Beyond ADP-ribosylation of thymine with DarT2, a large number of base-modifying enzymatic domains against any of the four nucleotides could expand append editing. For instance, DarT1 toxins (related to DarT2) and eukaryotic toxins called pierisins (found in cabbage moths) ADP-ribosylate the N2 position of guanine^48,49^, with evidence of base mutagenesis by pierisins in Chinese hamster ovary cells^50^. Additionally, bacteria and bacteriophages append unique chemical moieties such as methylcarbamoyl^51^, dPreQ_0_ (ref. ^52^), dADG^53^, glucosyl-5-hydroxymethyl^54^ and 5-hydroxymethyl^55^ to their DNA to block access by antiphage defenses^56^. The associated enzymatic domains could be further engineered to alter the modified nucleotide, the recognized motif or the appended moiety and enhance editing efficiencies. Interestingly, these examples consistently derive from host–pathogen or host–parasite interactions that could serve as a plentiful source of such base-modifying domains.

Lastly, apart from genome editing, appending chemical moieties to DNA in a targeted manner could facilitate the study of localized versus genome-wide DNA repair. Evaluating the impact of DNA adducts is central to elucidating responsible modes of repair potentially driving mutagenesis and carcinogenesis. To date, introducing such adducts at specific chromosomal sites has proven extremely difficult and laborious^57^. With append editing, specific adducts could be studied in real time^58^ or in conjunction with genome-wide screening of repair pathways^59^, thus uncovering the molecular basis of editing outcomes and probable strategies to shape these outcomes.

## Methods

### Polymerase-blocking assays

WT and inactivated (E170A) EPEC DarT2 proteins were expressed using the cell-free myTXTL master mix (Arbor Biosciences). Linear DarT expression templates were amplified from plasmids or ordered as synthetic gene fragments (Integrated DNA Technologies) and contained a T7 promoter and a T7 terminator (Supplementary Table 2). Cell-free expression was performed in 12-µl reactions, comprising 9 µl of myTXTL master mix, 4 nM of EPEC DarT2 template, 0.4 nM of a T7 RNA polymerase-encoding plasmid and 4 µM of the RecBCD inhibitor GamS to prevent degradation of the linear DNA templates. The reactions were incubated for 16 h at 29 °C.

For ADP-ribosylation of ssDNA templates, the ADP-ribosylation assay was adapted from prior work with slight alterations^13^. Briefly, 5 µl of the TXTL reaction mix was incubated with 10 µM of the ssDNA oligo, 50 µM NAD^+^, 50 mM Tris-HCl pH 8, 150 mM NaCl, 10 mM EDTA and sterile nuclease-free water to reach a final volume of 20 µl and incubated for 30 min at 30 °C. Afterward, the oligos were separated from the mix using the Oligo clean and concentrator kit (Zymo).

To assess whether DNA ADP-ribosylation blocks DNA polymerases in vitro, the DarT-treated oligos were first annealed to the 5′ 6-Fam-tagged primer CKo20 at a final concentration of 10 µM in 1× NEBuffer 2 by heating the mixture to 94 °C and gradually cooling it to room temperature. Next, 2 µl of the annealed product was mixed with 0.5 U of Klenow fragment (New England Biolabs), 33 µM dNTPs and 1× NEBuffer 2 in a total volume of 12.5 µl and incubated for 15 min at 37 °C. To stop the reaction, EDTA was added to a final concentration of 10 mM and the samples were incubated at 75 °C for 20 min.

To visualize the block of polymerization, 4 µl of the polymerization product was mixed with 4 µl of loading dye (containing 95% formamide, 0.03% SDS, 18 mM EDTA, 23 µM xylene cyanol and 19 µM bromophenol blue) and loaded onto a preheated denaturing polyacrylamide gel (8 M urea and 20% polyacrylamide (19:1)). The gel was run at 250 V for 30 min and visualized under ultraviolet light before and after staining with SYBR gold (Thermo Fisher).

### Microbial strains, handling and growth conditions

All bacterial and yeast strains used in this study are listed in Supplementary Table 2. Unless otherwise specified, *E.* *coli* TOP10 was used for plasmid cloning and propagation and was grown at 37 °C in Luria–Bertani (LB) liquid medium (10 g L^−1^ tryptone, 5 g L^−1^ yeast extract and 10 g L^−1^ NaCl) shaking orbitally at 200 rpm or on LB solid medium (15 g L^−1^ agar) at 37 °C, containing kanamycin (50 mg L^−1^), carbenicillin (100 mg L^−1^) or chloramphenicol (34 mg L^−1^), when appropriate. The *E.* coli kanR* strain (CBS-4802) began as strain CB330 (*E.* *coli* MG1655 P_J23110_-*araFGH* ∆*araBAD*), selected for uniform arabinose induction, to which two chromosomal modifications were made. First, the ∆*lacZ* phenotype (W519*) was generated by CBE-mediated deamination of 5′-ACC-3′ to 5′-ATT-3′ (positions 364,749 and 364,750 in MG1655), resulting in a premature stop codon; this edit was not used in this work. Second, a defective *kanR* expression construct (kanR*) (annotated sequence of the genomic locus in Supplementary Table 2) containing a premature stop codon (Q177*) and DarT2 motif 5′-TTTC-3′ was inserted between genes *ybjM* and *grxA* (positions 890,463–890,480 in MG1655) by Red-mediated recombination with Cas9 counterselection^1,36,65^. The resulting *E.* *coli* MG1655 kanR* strain was used for all assays related to the kanR* gene. The kanR* strain was further used to generate Δ*recA*, Δ*recB*, Δ*recF*, Δ*recT*, Δ*recJ*, Δ*recO*, Δ*xthA*, Δ*mutS* and Δ*uvrA* mutants by Red-mediated recombination^66^. Briefly, transformants of the *E.* coli kanR* strain carrying pKD46 (encoding λ Red-γ, Red-β and Red-exo) were cultured in l-arabinose at 30 °C until an optical density at 600 nm (OD_600_) of ~0.6, made electrocompetent as previously described^66^ and then transformed with a linear dsDNA template containing 40-nt homology arms to mediate deletion of the target gene. Next, pKD46 was cured from the bacteria by growing them at 37 °C, after which the bacteria were made electrocompetent and transformed with pCP20 and then grown at 42 °C to simultaneously express FLP recombinase and eliminate pCP20. Colonies were then screened for gene deletion by colony PCR and Sanger sequencing. For the substitution assays targeting the *aaaD*, *punR*, *ygcQ* and *yheO* genes, the *E.* *coli* MG1655 strain was used.

*Salmonella enterica* subsp. *enterica* serovar Typhimurium strain LT2 was used for all ADPr-TAE assays in *Salmonella* and was regularly grown at 37 °C in LB liquid medium shaking orbitally at 200 rpm or on solid LB medium. Carbenicillin (100 mg L^−1^) and chloramphenicol (34 mg L^−1^) were supplemented in the growth medium when necessary.

The *S.* *cerevisiae* BY4741 (Δ*trp1*, Δ*leu2*) strain was used for all yeast experiments. Unless otherwise specified, *S.* *cerevisiae* was grown in nonselective liquid YPD medium (20 g L^−1^ peptone, 10 g L^−1^ yeast extract and 2% (w/v) d(+)-glucose) or on solid nonselective YPD medium (20 g L^−1^ agar). To select for transformants, *S.* *cerevisiae* cells were grown on solid synthetic defined (SD) medium without tryptophan and leucine, containing 6.9 g L^−1^ yeast nitrogen base without amino acids (Formedium, CYN0402), 0.64 g L^−1^ complete supplement mixture without tryptophan and leucine (Formedium, DCS0569), 20 g L^−1^ d(+)-galactose (Sigma-Aldrich, 15522-250G-R) and 20 g L^−1^ agar (Th. Geyer, 214510).

### Plasmid construction

Annotated sequences of all plasmids used in this study are provided in Supplementary Table 2. Unless otherwise specified, general cloning methods such as KLD (KLD enzyme mix, M0554S) or Gibson assembly (NEBuilder HiFi DNA assembly master mix, E2621X) were used to assemble linear dsDNA fragments into plasmids. Linear dsDNA fragments were amplified with Q5 high-fidelity 2× master mix (New England Biolabs, M0492L) and purified using the NucleoSpin gel and PCR cleanup kit (Macherey-Nagel, 740609.50). Plasmid sequences were verified by full plasmid sequencing (Plasmidsaurus) or Sanger sequencing (Microsynth Seqlab).

To generate the append editors expressed in plants, the codon-optimized DNA sequence for DarT2^D^ was commercially synthesized (Twist Bioscience) with a previously reported N7-NLS for expression in *N.* *benthamiana*^67^, while the zCas9i (*Zea mays* codon-optimized Cas9 coding sequence with 13 introns) was obtained from Addgene (kit 1000000171)^68^. Both fragments were amplified using the iProof high-fidelity PCR kit (Bio-Rad, 1725331). The dDarT, nzCas9i and dzCas9i variants were generated using inverse PCR. Three gRNAs targeting the phytoene desaturase 1 gene (*PDS1*) (Supplementary Table 2) were cloned by annealing complementary oligos into an AtU6 gRNA cassette. Gene fragments were assembled using the GoldenBraid cloning strategy^69^.

### kanR* reversion

To assess ADPr-TAE in *E.* *coli*, an overnight culture of strain CBS-4802 was backdiluted 100-fold, grown to an OD_600_ of 0.6–0.8 and then rendered electrocompetent in 10% glycerol. For transformation, 40 μl of electrocompetent cells were mixed with 9 fmol of the relevant plasmid(s) and transferred to an ice cold 1-mm electroporation cuvette (Bio-Rad Laboratories, 1652089). Cells were electroporated using the GenePulser Xcell microbial system (Bio-Rad Laboratories, 1652662) and the following settings: 1.8 kV, 25 µF and 200 Ω. Next, cells were supplemented with 500 μl of SOC medium (5 g L^−1^ yeast extract, 20 g L^−1^ tryptone, 0.584 g L^−1^ NaCl, 0.186 g L^−1^ KCl, 2.4 g L^−1^ MgSO_4_ and 20 mM glucose) and recovered for 1 h at 37 °C, shaking orbitally at 200 rpm. Cells were collected by centrifugation at 3,000*g*, the supernatant was decanted and cells were resuspended in 2 ml of induction medium (LB, l-arabinose (0.2% w/v), carbenicillin (100 mg L^−1^) and chloramphenicol (34 mg L^−1^)) and incubated at 37 °C for 16 h, shaking orbitally at 200 rpm. Afterward, cell cultures were serially diluted in five tenfold steps in LB, from which 3 μl of each dilution was spotted on LB solid medium containing either carbenicillin and chloramphenicol to select for transformed cells or carbenicillin, chloramphenicol and kanamycin to select for transformed and edited cells. The spotted LB solid medium was then incubated for 16 h at 37 °C followed by counting colonies.

### Replacement, deletion and insertion assays in *E.coli*

For the *E.* *coli* replacement, deletion and insertion assays at the kanR* locus and the substitution assays at the *aaaD*, *punR*, *ygcQ* and *yheO* genes, an identical transformation and selection protocol was used as described above. However, after the 16-h incubation in the induction medium, 100 μl of the cell culture was plated on LB solid medium containing carbenicillin and chloramphenicol to obtain single colonies. Single colonies were resuspended in Q5 high-fidelity 2× master mix containing the appropriate primers and subjected to PCR amplification following the instructions of the manufacturer and extending the initial heating step of 98 °C to 5 min to mediate cell lysis and release of genomic DNA. Amplicons were purified and sequenced through Sanger sequencing.

### Growth-based toxicity assay in *E.coli*

The growth-based toxicity assay began by rendering strain CBS-5301 electrocompetent. Next, 9 fmol of plasmid CBS-4808 was transformed into strain CBS-5301 using the electroporation conditions described above. Transformants were recovered in 500 µl of SOC medium for 1 h at 37 °C, shaking orbitally at 200 rpm, then plated on LB solid medium supplemented with carbenicillin and incubated for 16 h at 37 °C. Next, a single colony was inoculated into 2 ml of LB medium containing carbenicillin, grown until an OD_600_ of 0.6 and then made electrocompetent following the protocols described above. A second round of transformation was performed, using one of nine different editor plasmids (CBS-6738, CBS-6739, CBS-6741, CBS-6742, CBS-6743, CBS-6744, CBS-6745, CBS-4781 or CBS-4800), following the electroporation protocol described above. Transformed cells were allowed to recover in 500 µl of SOC medium for 1 h at 37 °C shaking orbitally at 200 rpm, plated on LB solid medium supplemented with carbenicillin, chloramphenicol and glucose (20 mM) and incubated for 16 h at 37 °C. Three individual colonies from each of the nine resulting strains (Supplementary Table 2) were then used to inoculate a 96-deep-well plate (Greiner Bio-One, 780271), containing 400 µl of LB medium supplemented with carbenicillin, chloramphenicol and glucose (20 mM) and covered with an adhesive gas-permeable membrane (Thermo Scientific, 241205). After incubating the deep-well plate for 16 h at 37 °C, the cell cultures were adjusted to an OD_600_ of 0.1 using LB supplemented with carbenicillin, chloramphenicol and l-arabinose (0.2% w/v) in a new 96-well plate, reaching a final volume of 200 µl. The 96-well plate was then measured every 3 min over 12 h at 37 °C for absorbance at 600 nm on a BioTek Synergy Neo2 plate reader, shaking at 500 rpm.

### Nonselective editing at kanR*

Transformations were performed as described above; however, after the 16-h incubation in induction medium, the cultures were centrifuged, the medium was discarded and genomic DNA was isolated using the Wizard gDNA purification kit (Promega, A1120). The kanR site was then amplified through PCR using the primer pair HBo-314 and HBo-315 and the Q5 high-fidelity 2× master mix for 25 cycles. Resulting amplicons were sequenced with Nanopore sequencing (Eurofins Genomics). For data analysis, FASTQ sequencing data files were aligned to a FASTA file of the unedited amplicon using MiniMap2 with option ‘map-ont’^70^. SAMtools was used to convert the SAM files into BAM files, while concurrently sorting and indexing^71^. All further analysis was performed using R, after calling libraries tidyverse and GenomicAlignments^72^. A function was defined to take BAM files as an argument and then extract all alleles aligned to the 8-nt region of the templated edit as a list of characters. This function was applied to all BAM files to generate lists of alleles, which were tallied and compiled into a single data frame in long table format. Next, alleles were defined as unedited, edited or ambiguous and the fraction of each observation was computed. Samples were then grouped by editor and repair plasmids, after which the mean and s.d. were computed and then used to generate the bar plot. Further analysis was undertaken to search for base mutations at the ADPr site. The list of alleles in the initial data frame was filtered to retain only records containing a T-to-V mutation at the ADPr target position but otherwise matching the reference allele. Records were grouped by sample and SNVs were tallied, after which each was divided by the total number of observed alleles and multiplied by 100 to obtain the percentage of base mutations amongst all sequencing reads.

### Whole-genome off-target assay in *E.coli*

For identifying whole-genome off-target mutations, strain CBS-4802 was grown from a single colony in LB medium and made electrocompetent as described above. Electrocompetent CBS-4802 was then cotransformed with equimolar amounts (9 fmol) of CBS-6746 and one of several editor plasmids (CBS-3130, CBS-6738 or CBS-6740). Transformants were recovered in 500 μl of SOC for 1 h at 37 °C shaking orbitally at 200 rpm, after which the growth medium was replaced with 2 ml of LB, supplemented with carbenicillin, chloramphenicol and l-arabinose (0.2%), followed by incubation at 37 °C for 16 h shaking orbitally at 200 rpm. Next, the cultures were streaked onto LB solid medium supplemented with carbenicillin and chloramphenicol and incubated for 16 h at 37 °C to obtain individual colonies. Three colonies from each condition were placed in 2 ml of LB medium supplemented with carbenicillin and chloramphenicol and cultured for 16 h at 37 °C.

After incubation, cultures were centrifuged and the cell pellets were subjected to genomic DNA isolation using the Wizard genomic DNA purification kit. Isolated genomic DNA was fully sequenced using Nanopore sequencing (Plasmidsaurus). For data analysis, FASTQ sequencing data files were aligned to a FASTA file of *E.* *coli* MG1655 (GenBank: U00096.3) using Minimap2 with the ‘map-ont’ option^70^. SAMtools was used to convert the SAM files into BAM files, while concurrently sorting and indexing^71^. Clair3 was run on the GalaxyEU server to call variants^73,74^. Bcftools was used to query the VCF files for POS, REF, ALT, DP and AF fields and export the results into a CSV file^75^. The sequencing depth at all positions in all BAM files was calculated by SAMtools and exported as a CSV file. All further analysis was performed in R after loading library tidyverse^72^. CSV files were loaded into a long-format data frame. This data frame was then filtered as follows: (1) SNVs were retained by filtering for records that contain only a single character in the REF and ALT fields; (2) SNVs already present in the parent strain were eliminated by filtering for records containing POS field values not found in parent strain POS field values; (3) SNVs mapped to regions known to have been modified during the creation of strain CBS-4802 were eliminated by filtering for records with POS field values not present in said regions; (4) records were filtered for AF field values greater than or equal to 0.25; (5) SNVs observed at a sequencing depth greater than or equal to the lowest quartile of all BAM files (Q1 ≥ 34) were retained; and (6) all SNVs were recoded to C > D and T > V, tallied and then used to generate a heat map.

### Editing assays in *S.enterica*

Electrocompetent *S.* *enterica* cells were transformed with 9 fmol of plasmid CBS-4800 and recovered in 500 μl of SOC medium following an identical protocol to that described above for *E.* *coli*. After recovery, the cells were collected through centrifugation at 3,000*g*, the supernatant was decanted and the cell pellet was resuspended in 100 μl of LB medium. The cell suspension was plated on LB solid medium containing chloramphenicol (34 mg L^−1^) and incubated at 37 °C for 16 h. After incubation, a single colony was selected and used to create electrocompetent *S.* *enterica* cells harboring plasmid CBS-4800 following the protocol described above. Then, 22 fmol of the plasmids containing the repair template and the T sgRNA (Supplementary Table 2) were transformed in triplicate through electroporation into *S.* *enterica* cells harboring plasmid CBS-4800. The cells were recovered in 500 μl of SOC medium and collected through centrifugation at 3,000*g*, the supernatant was decanted and the cell pellet was resuspended in 2 ml of induction medium (LB, 0.2% (w/v) l-arabinose, 100 mg L^−1^ carbenicillin and 34 mg L^−1^ chloramphenicol) and grown at 37 °C for 16 h, shaking orbitally at 200 rpm. Next, 100 μl of the cell culture was plated on LB solid medium containing carbenicillin and chloramphenicol to obtain single colonies. Colonies were resuspended in Q5 high-fidelity 2× master mix containing the appropriate primers and subjected to PCR amplification following the instructions of the manufacturer and adding an initial heating step of 98 °C for 5 min to mediate cell lysis and release of genomic DNA. Amplicons were then purified using the NucleoSpin gel and PCR cleanup kit and sequenced through Sanger sequencing.

### Templated editing assays in *S.cerevisiae*

*S.* *cerevisiae* BY4741 (Δ*trp1*, Δ*leu2*) cells were cotransformed with two plasmids, one bearing the specified editor variant and the other bearing a 6-bp substitution template flanked by 294-bp (upstream) and 232-bp (downstream) homology arms along with an *FCY1* T sgRNA or NT sgRNA (Supplementary Table 2), following the lithium acetate method as previously described^76^.

Briefly, single *S.* *cerevisiae* colonies were inoculated into 2 ml of liquid YPD medium (20 g L^−1^ peptone, 10 g L^−1^ yeast extract and 2% (w/v) d(+)-glucose) and grown for 16 h at 30 °C, shaking at 200 rpm on a rotary shaker. The cells were diluted to an OD_600_ of 0.5 in 50 ml of YPD medium and cultured again at 30 °C, shaking at 200 rpm, until the cells reached an OD_600_ of 2. The cells were then harvested by centrifugation at 3,000*g* for 5 min, the supernatant was decanted and the pellet was resuspended in 25 mL of sterile water. The centrifugation and resuspension step was repeated followed by another centrifugation at 3,000*g* for 5 min and resuspension in 1 ml of sterile water. The cell suspension was then centrifuged for 30 s at 13,000*g*, the supernatant was discarded and the pellet was resuspended in 1 ml of sterile water. Next, 100-μl aliquots were distributed in 1.5-ml sterile Eppendorf tubes and the cells were collected by centrifugation at 13,000*g* for 30 s. The supernatant was decanted and the cell pellet was resuspended with 326 μl of transformation mix (240 μl of PEG 3350, 36 μl of 1 M lithium acetate and 50 μl of 2 mg ml^−1^ carrier ssDNA), plasmid DNA (500 ng of each plasmid) and sterile water to reach a final volume of 360 μl. The suspension was incubated at 42 °C for 40 min, after which it was centrifuged at 13,000*g* for 30 s. The supernatant was decanted, the cell pellet was resuspended in 1 ml of YPD medium and the cell suspension was incubated for 3 h at 30 °C. Cells were collected by centrifugation at 13,000*g* for 30 s and washed twice with 1 ml of SD medium to remove any residual YPD medium. Finally, the cell pellet was resuspended with 100 μl of SD medium, plated on solid SD medium without tryptophan and leucine and containing d-galactose and incubated at 30 °C for 3 days or until colonies were visible.

Resulting colonies were collected with a sterile 10-μl pipette tip and resuspended in 10 μl of sterile 0.02 M NaOH, boiled at 99 °C for 10 min and centrifuged for 10 s at maximum speed in a microcentrifuge. Then, 1 μl of the supernatant was used as template for PCR using the Q5 high-fidelity 2× master mix and the primer pair prCP222–prCP223 to amplify *FCY1* (Supplementary Table 2). The resulting PCR product was purified using the NucleoSpin gel and PCR cleanup kit, following the manufacturer’s instructions. The final product was sequenced through Sanger sequencing. Sequence alignment was performed using the online MAFFT algorithm^77^.

### Base mutation assays in *S.cerevisiae*

*S.* *cerevisiae* BY4741 (Δ*trp1*, Δ*leu2*) cells were cotransformed with two plasmids, one bearing the specified editor variant and the other bearing either of the T sgRNAs for *FCY1*, *ALP1* or *JSN1* or an NT sgRNA (Supplementary Table 2), following identical procedures to those described above. Resulting colonies were screened through colony PCR as described above and the primer pairs prCP222–prCP223, prCP445–prCP446 and prCP441–prCP442 were used to amplify *FCY1*, *ALP1* and *JSN1*, respectively (Supplementary Table 2). The resulting PCR products were sequenced through Sanger sequencing and sequence alignment was performed using the MAFFT algorithm^77^.

### Base mutation assays in *N.benthamiana*

*N.* *benthamiana* seeds were germinated in soil and transplanted at the 1-week-old stage to 24 cell nursery flats, one plant per cell, and grown at 23 °C under a 16-h light and 8-h dark cycle in Sungro horticulture professional grow mix mixed 1:1 with Jolly gardener Pro-line C/B growing mix (Sungro).

Plasmids were used to electroporate *Agrobacterium tumefaciens* strain GV3101 using Bio-Rad GenePulser electroporator with the following conditions: 1.8 kV, 100 Ω and 25 µF. Single colonies were inoculated in LB medium containing spectinomycin (100 µg ml^−1^), rifampicin (50 µg ml^−1^) and gentamicin (50 µg ml^−1^) for 16 h at 28 °C with orbital shaking at 200 rpm. Cultures were then centrifuged and resuspended in infiltration medium (10 mM MgCl_2_ and 100 µM acetosyringone) to reach an OD_600_ of ~0.1. Next, the resuspended cultures were combined in a 1:1 ratio with an *A.* *tumefaciens* strain containing *p19* (a suppressor of gene silencing) and were infiltrated into the leaves of 4-week-old plants using a 1-ml needleless syringe. The infiltrated plants were then recovered overnight in the dark and grown for 7 days using the conditions mentioned above.

### Next-generation sequencing in *N.benthamiana*

Leaf tissues were isolated 7 days after infiltration using a standard hole punch and collected in 1.5-ml tubes containing ~100 µl of 1 mm glass beads. Disks from four leaves (one disk per leaf) were pooled to create each biological replicate. The samples were frozen at −80 °C for 24 h, after which the tissue was ground using a Vivadent shaker for 5 s followed by resuspension in CTAB buffer (1.4 M NaCl, 20 mM EDTA pH 8, 100 mM Tris-HCl pH 8 and 3% CTAB). Cellular DNA was then extracted using chloroform and isopropyl alcohol followed by a 70% ethanol wash.

The targeted region was amplified with optimized primers and PCR conditions, using an iProof high-fidelity PCR kit. The products were purified using 4 µl of ExoSAP-IT PCR product cleanup reagent (Applied Biosystems, A55242) at 37 °C for 15 min followed by inactivation at 80 °C for 15 min. A second amplification was performed with iProof polymerases to introduce unique Illumina barcodes and libraries were purified using the QIAquick gel extraction kit (Qiagen).

The concentration for each library was measured using Qubit fluorometer (Invitrogen) and equimolar amounts were pooled along with the 120 pM phiX control library corresponding to 8% of the final volume. Then, 20 μl of the pooled library was loaded into the iSeq 100 (Illumina) and the run was performed in accordance with iSeq 100 sequencing system guide. Sequencing data analysis was performed as mentioned for mammalian cells.

### Mammalian cell culture and transfection

HEK293T cells were purchased from the American Type Culture Collection (CRL 11268) and U2OS^Δ*TARG1*^ cell lines were a gift from the I. Ahel lab. Unless otherwise mentioned, all cell lines were maintained using DMEM (Life Technologies) supplemented with 10% (v/v) FBS (Corning and BANF Biotrend), 1× penicillin–streptomycin (Life Technologies) and 2 mM l-glutamine. The cultures were incubated in humidified incubators at 37 °C with 5% CO_2_.

For generating the HEK293T Δ*TARG1* cell line, cells were transfected with plasmids containing WT SpCas9 and *TARG1* sgRNA^28^ (Supplementary Table 2) using Lipofectamine 3000 (Invitrogen, L3000008) according to the manufacturer’s instructions. Then, 48 h after transfection, cells were diluted and seeded in 96-well plates at a density of three cells per well. Colonies were observed after 7 days and wells with single colonies were selected. Selected clones were tested for *TARG1* site disruption through Sanger sequencing followed by western blotting (Supplementary Fig. 8) with anti-*TARG1* antibody (Fisher Scientific, 25249-1-AP)^28^ and anti-β-actin antibody (Life Technologies, MA5-15739-HRP) as the housekeeping control.

For templated editing assays in HEK293T (WT and Δ*TARG1*) cell line, 65,000 cells per well were seeded onto tissue-culture-treated 24-well plates (Corning) and incubated at 37 °C with 5% CO_2_ under humidified conditions. Then, 24 h later, 50 fmol of each plasmid was cotransfected with 750 fmol of single-stranded oligodeoxynucleotide repair templates using 1.12 μl of Lipofectamine 3000 reagent and 1 μl of P3000. For base mutagenesis assays, 500 ng of each plasmid was transfected, following the same conditions as mentioned above. The medium was refreshed 24 h after transfection and cells were collected 72 h after transfection.

For base mutagenesis assays in the U2OS^Δ*TARG1*^ cell line, 1.3 × 10^5^ cells were seeded and 1 μg of plasmid DNA, 1.5 μl of Lipofectamine 3000 reagent and 2 μl of P3000 were used for transfection. Medium change and sample collection were performed similarly to HEK293T cells.

For orthogonal R-loop assays in HEK293T^Δ*TARG1*^ cell lines, 65,000 cells were seeded per well in 24-well plates and cotransfected after 24 h with 300 ng of SpCas9-based editor plasmids, 200 ng of SpCas9 guide plasmid, 300 ng of dSaCas9 plasmid (Addgene, 138162) and 200 ng of SaCas9 guide plasmid. Then, 1.5 µl of Lipofectamine 3000 and 2 µl of P3000 reagent were used for transfection; cell pellets were collected after 72 h.

### RNA interference

For the RNA interference experiments, Dicer-substrate siRNAs were designed and purchased from Integrated DNA Technologies (TriFECTa RNAi Kit, design ID: hs.Ri.OARD1.13). All siRNA transfections were performed in HEK293T cells using Lipofectamine RNAiMAX (Invitrogen, 13778075) according to the manufacturer’s instructions. A total of 80,000 cells were seeded per well in tissue-culture-treated 24-well plates (Corning) and forward-transfected with 10 nM siRNA. After 48 h, 500 ng of each plasmid was transfected under the same conditions as described above. The medium was refreshed 24 h after plasmid transfection and cells were harvested 72 h after plasmid transfection.

The knockdown efficiency of *TARG1* expression was assessed at the transcript level by real-time qPCR. Briefly, 80,000 cells were seeded and transfected with 10 nM siRNA and total RNA was extracted after 72 h using TRIzol reagent (Invitrogen) according to the manufacturer’s protocol. RNA (500 ng) was used for one-step real-time qPCR using the iTaq Universal SYBR green one-step kit (Bio-Rad, 172-5151) on a CFX96 real-time PCR detection system (Bio-Rad). The thermal cycling conditions were as follows: 50 °C for 10 min (reverse transcription), 95 °C for 1 min, followed by 40 cycles of 95 °C for 10 s and 60 °C for 30 s and a final melt curve analysis. The following primers were used for real-time qPCR: TARG1 forward, 5′-AAAGGAGACCTTTTTGCAT-3′; TARG1 reverse, 5′-GATTTAAAAGTTCTTGCACCC-3′. For each biological replicate, mRNA levels were quantified using the *ΔΔC*_*t*_ method, with normalization to HPRT expression and comparison to the corresponding NT siRNA control. Final values represent the mean relative expression across biological replicates.

### Next-generation sequencing for mammalian cells

Genomic DNA was isolated from harvested cells using PureLink genomic DNA mini kit (Life Technologies, K182002). Specific primers were used to amplify the targeted region using Q5 high-fidelity 2× master mix through 27 cycles. The PCR product was purified using the NucleoSpin gel and PCR cleanup kit and was used as a template in KAPA HiFi HotStart ReadyMix (Roche Diagnostics, KK2602) to introduce Illumina adaptor sequences within 15 PCR cycles. The KAPA-PCR products were cleaned using Agencourt AMPure XP magnetic beads (Beckman Coulter, A63881) and 200 ng of this product was used as template for a second PCR with KAPA ReadyMix to introduce Illumina barcodes through ten PCR cycles followed by cleanup using magnetic beads as mentioned before. PCR products were screened at each step for correct fragment length using agarose gel electrophoresis. The libraries were pooled in equimolar amounts and at least 1 million reads were generated for each sample using NovaSeq 6000 and NextSeq 2000. The demultiplexed data were analyzed using CRISPResso2 (ref. ^78^). Default parameters were used to perform the analysis except when quantifying indel and HDR frequencies for templated editing, in which case a plot window size of 30 was used. Allelle_frequency_table_around_sgRNA.txt files generated by CRISPResso2 were used within R scripts (https://github.com/saliba-lab/ADPr_TAE_analysis) to further quantify base mutation frequencies as the total percentage of reads containing a nucleotide different from the reference read.

### Nanopore sequencing

The following steps were carried out in an amplicon-free pre-PCR area. First, 500 ng of genomic DNA was amplified using NEBNext Ultra II Q5 HiFi polymerase (New England Biolabs) with primers containing stubbers for downstream indexing. The expected amplicon length was 4.4 kb surrounding the cut site. The following PCR cycle conditions were used: denaturation at 98 °C for 30 s, followed by 25 cycles of 98 °C for 10 s, 60 °C for 30 s and 72 °C for 5 min. PCR products were purified with 0.8× solid-phase reversible immobilization beads and eluted in H_2_O. Libraries were indexed and generated using the PCR barcoding expansion 1–96 (EXP-PBC096) for ligation sequencing kit (SQK-LSK114, Oxford Nanopore). Purified libraries were sequenced on a PromethION with the R10.4.1 flow cell. Read lengths were quantified using SummarizeOntDels (https://github.com/cornlab/summarizeOntDeletions)^32^.

### Statistical analyses

For assays involving *kanR* reversion on solid medium (Figs. 1g and 2b), unpaired, two-tailed Welch’s *t*-tests were performed on log-normal data. Figure error bars display the s.d. For the nonselective editing experiment (Supplementary Fig. 3), a one-way analysis of variance was performed to test for the effect of editor–sgRNA combinations on the percentage of reads showing an SNV at the target thymidine. For the assay involving deletion strains in *E.* *coli* (Fig. 2e), unpaired, one-tailed Welch’s *t*-tests were performed on log-normal data. Figure error bars display the s.d. For short-read next-generation sequencing data (Figs. 3f,g and 4c–e,g), unpaired, two-tailed Welch’s *t*-tests were performed. Figure error bars display the s.e.m. For the editing window experiment (Fig. 4f), the median and quartiles of each group are displayed. Related *P*-value calculations can be found in the Source Data and Supplementary Data 1.

### Reporting summary

Further information on research design is available in the Nature Portfolio Reporting Summary linked to this article.

## Online content

Any methods, additional references, Nature Portfolio reporting summaries, source data, extended data, supplementary information, acknowledgements, peer review information; details of author contributions and competing interests; and statements of data and code availability are available at 10.1038/s41587-025-02802-w.

## Supplementary information

Supplementary Information Supplementary Figs. 1–18, Table 1 and references.

Reporting Summary

Supplementary Table 2 Strains, plasmids, oligos, sgRNA guides and siRNAs used in this work.

Supplementary Table 3 Potential disease targets for ADPr-TAE.

Supplementary Data 1 Source data for supplementary figures.

## Source data

Source Data Fig. 1 Statistical source data.

Source Data Fig. 1 Unmodified gels.

Source Data Fig. 2 Statistical source data.

Source Data Fig. 3 Statistical source data.

Source Data Fig. 4 Statistical source data.

Source Data Fig. 5 Statistical source data.

Source Data Extended Data Fig. 1 Unmodified gel images.

Source Data Extended Data Fig. 3 Statistical source data.

Source Data Extended Data Fig. 7 Statistical source data.

Source Data Extended Data Fig. 8 Statistical source data.

Source Data Extended Data Fig. 9 Statistical source data.

Source Data Extended Data Fig. 10 Statistical source data.

## Data Availability

The high-throughput sequencing data were deposited to the National Center for Biotechnology Information under BioProject PRJNA1149814). The datasets generated for all figures are included in the Supplementary Information. There are no restrictions on data availability. Source data are provided with this paper.

## Code availability

R scripts used for the analysis of processed next-generation sequencing data were deposited to GitHub (https://github.com/saliba-lab/ADPr_TAE_analysis).
