# Supplementary material for: Targeted DNA ADP-ribosylation triggers templated repair in bacteria and base mutagenesis in eukaryotes
Source: Nat Biotechnol. 2025 Sep 4;44(7):1190–201. doi: 10.1038/s41587-025-02802-w (PMC13368585; doi:10.1038/s41587-025-02802-w)
Supplement: Supplementary file 1 — Supplementary Figs. 1–18, Table 1 and references. [file 41587_2025_2802_MOESM1_ESM.pdf]

nature biotechnology

Supplementary information

https://doi.org/10.1038/s41587-025-02802-w

Targeted DNA ADP-ribosylation triggers templated repair in bacteria and base mutagenesis in eukaryotes

In the format provided by the authors and unedited

Table of Contents

Supplementary Figures 2
Supplementary Tables 20
Supplementary References 22

SUPPLEMENTARY FIGURES

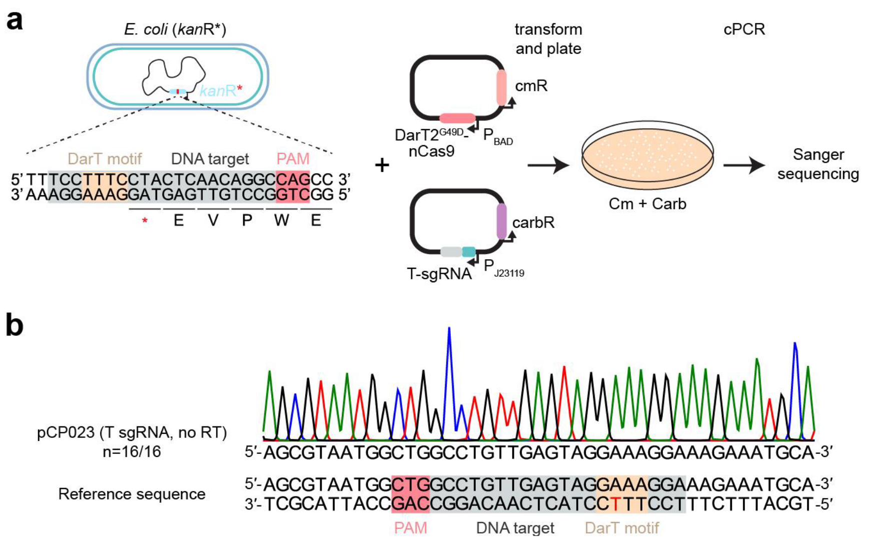

Supplementary Fig. 1: Representative genotype after targeting the *kanR site with DarT2^{D}-nScCas9 in the absence of a repair template. a.** Experimental setup for targeting the kanamycin resistance gene (*kanR**) in *E. coli* using the DarT2^{G49D}-nScCas9 editor and a targeting (T) sgRNA, without a repair template. **b**. Representative Sanger sequencing chromatogram of 16 sequenced colonies.

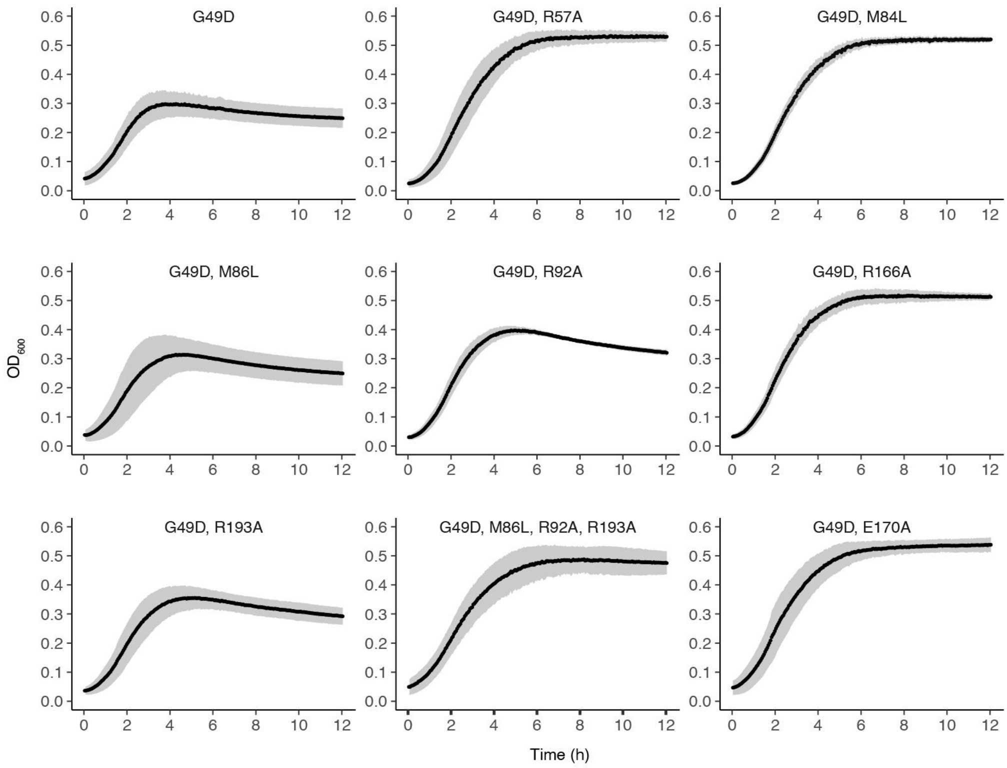

Supplementary Fig. 2: Growth curves of *E. coli* Δ*recA* strains transformed with different attenuated variants of DarT2 fused to nScCas9. Optical density measurements of cultures from a plate reader over the course of 12 hours, with absorbance measured at 600 nm and absorbance of the culture medium subtracted. Plots show the mean of triplicates in black, and the standard deviation of triplicates in grey.

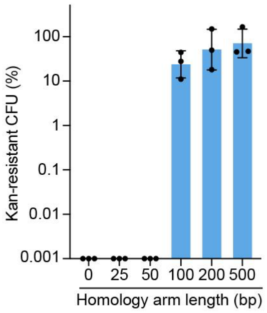

Supplementary Fig. 3: Impact of homology arm length on reverting *kanR with DarT2^{DLAA}-nScCas9 in *E. coli*.** The indicated lengths (in bp) were the same upstream and downstream of the 8-bp edit (e.g., 25 represents 25-bp homology upstream and 25-bp homology downstream of the edit). Bars and error bars represent the mean and s.d. of three independent experiments started from separate transformations.

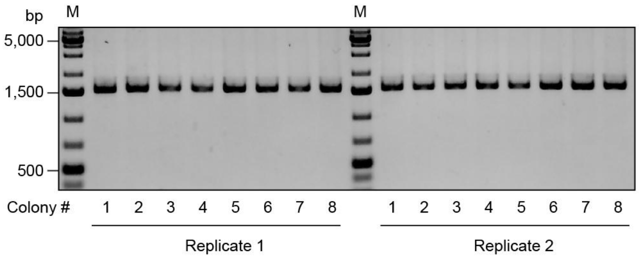

Supplementary Fig. 4: Colony PCR screening for 500-bp insertion at the *kanR target site in *E. coli* following targeting with DarT2^{DLAA}-nScCas9 in the presence of the repair template.** Two replicate transformations were used. Eight colonies from each replicate were screened through colony PCR using primers HBo312 and HBo313. M, marker. Results relate to the 500-bp insertion in Figure 2h.

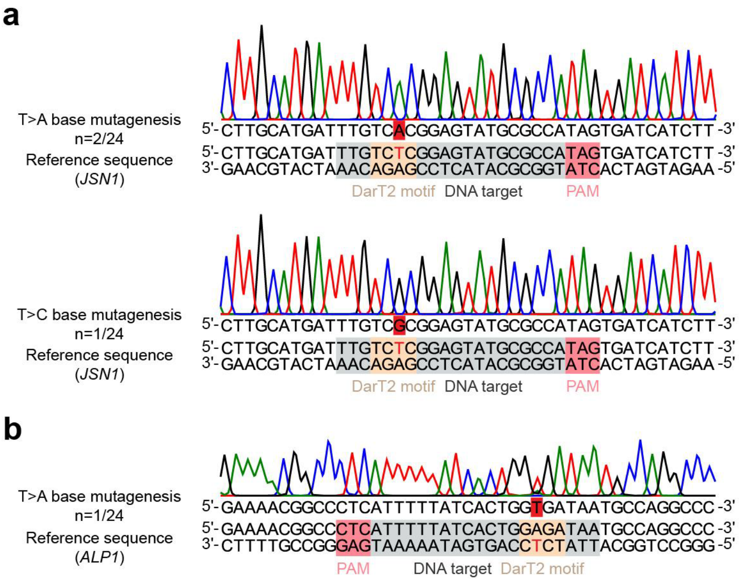

Supplementary Fig. 5: Representative genotypes after targeting the *JSN1 or ALP1* genes in *S. cerevisiae* with DarT^{DLAA}-nScCas9 in the absence of a repair template. a, Top: representative Sanger sequencing result showing T-to-A mutagenesis of the target thymine (labelled red) in the 5′-TYTN-3′ DarT2 motif. 24 colonies were screened, out of which two contained the T-to-A mutation. Bottom: representative Sanger sequencing result showing T-to-C mutagenesis of the target thymine (labelled red) in the 5′-TYTN-3′ DarT2 motif. 24 colonies were screened, out of which one contained the T-to-C mutation. **b,** Representative Sanger sequencing result showing T-to-A mutagenesis of the target thymine (labelled red) in the 5′-TYTN-3′ DarT2 motif. 24 colonies were screened, out of which one contained the T-to-A mutation. The mutated bases are highlighted in red.

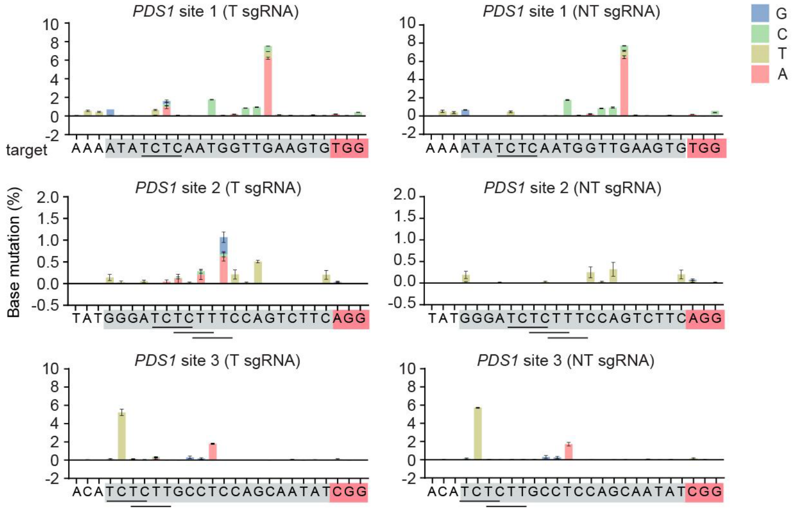

Supplementary Fig. 6: Location of base mutations with DarT2^{D}-nSpCas9 in the *PDS1* gene in *N. benthamiana* under targeting and non-targeting conditions. Bars and error bars represent the mean and SEM of three independent replicates without selection or sorting. Peaks observed with both targeting (T) and non-targeting (NT) sgRNAs could represent amplification of *PDS1* homeologs. To account for these peaks, values from NT were subtracted from those from the T sgRNA to generate the plots shown in Figure 3g.

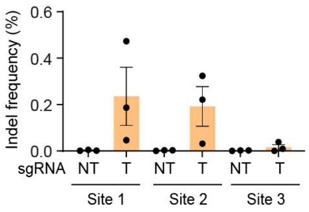

Supplementary Fig. 7: Frequency of indels generated with DarT2^{D}-nSpCas9 when targeting the *PDS1* gene of *N. benthamiana*. Bars and error bars represent the mean and SEM of three independent biological replicates, where each replicate consists of tissue from four infiltrated leaves. Dots represent measurements from individual samples. T sgRNA, targeting sgRNA. NT sgRNA, non-targeting sgRNA.

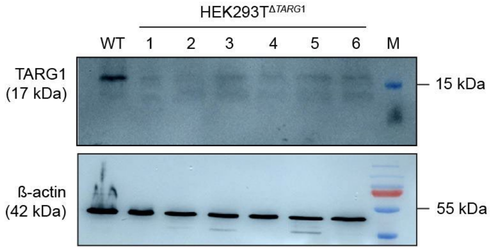

Supplementary Fig. 8: Confirming the *TARG1* knockout in HEK293T cell lines. Clones 1-6 demonstrated disruption at the TARG1 targeted site using Sanger sequencing. Loss of protein expression was validated via western blotting using antibodies specific to human TARG1 (ref. 1).

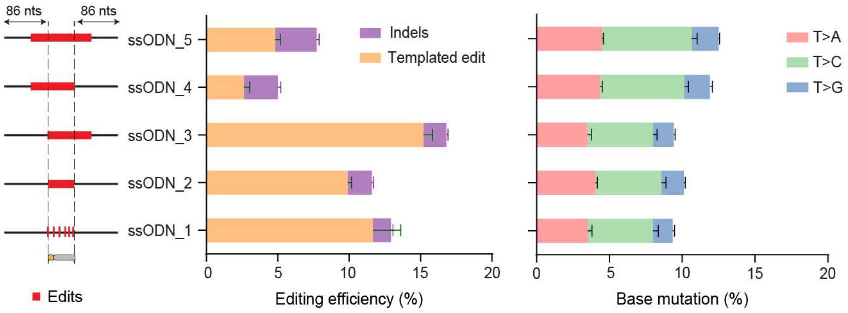

Supplementary Fig. 9: Base mutation with DarT2^{D}-nSpCas9 in HEK293T Δ*TARG1* cells using different single-stranded oligodeoxynucleotides (ssODN) repair templates. The editing assay was performed similar to Figure 4c. Bars and error bars represent the mean and SEM of three independent replicates without selection or sorting.

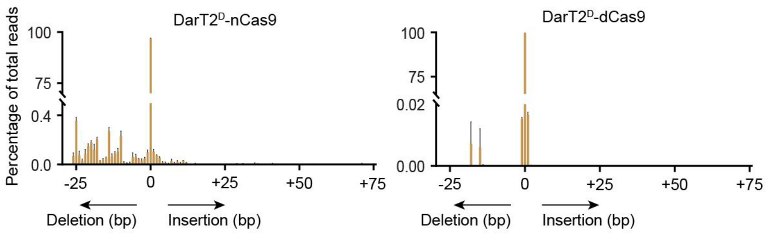

Supplementary Fig. 10: Size and frequency of indels when targeting the *EMX1* site 1 in HEK293T Δ*TARG1* cells with DarT2^{D}-nSpCas9. Values with '-' reflect size of deletions, '+' reflect size of insertions, while '0' represents the percentage of reads containing no insertions or deletions. Results derived from analysis of samples reported in Figure 4d. Bars and error bars represent the mean and SEM of three independent replicates without selection or sorting.

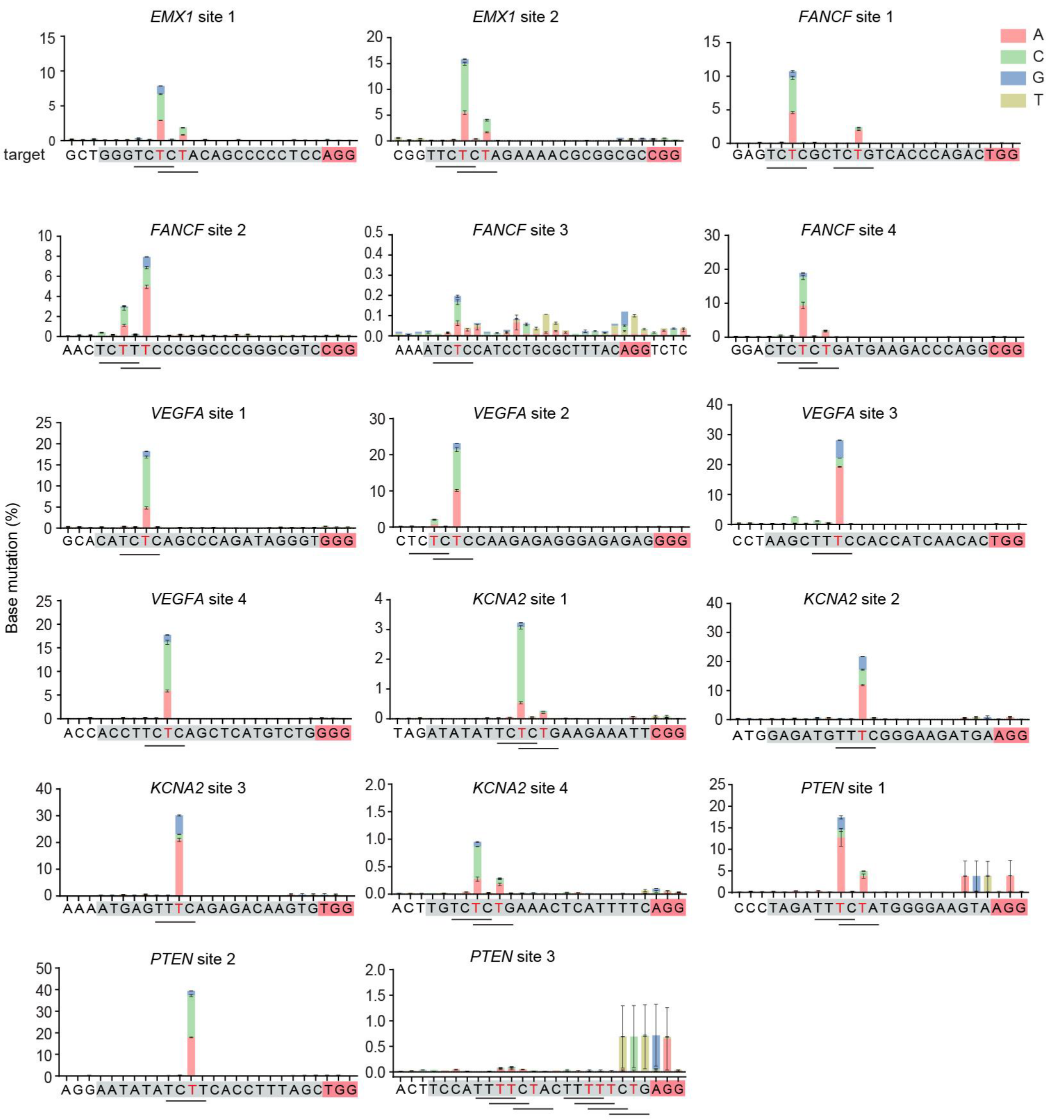

Supplementary Fig. 11: Location of base mutations with DarT2^{D}-nSpCas9 in HEK293T-Δ*TARG1* cells. Bars and error bars represent the mean and SEM of three independent replicates without selection or sorting. Horizontal bars indicate DarT2 recognition motifs, with ADP-ribosylation of the thymine at the third position.

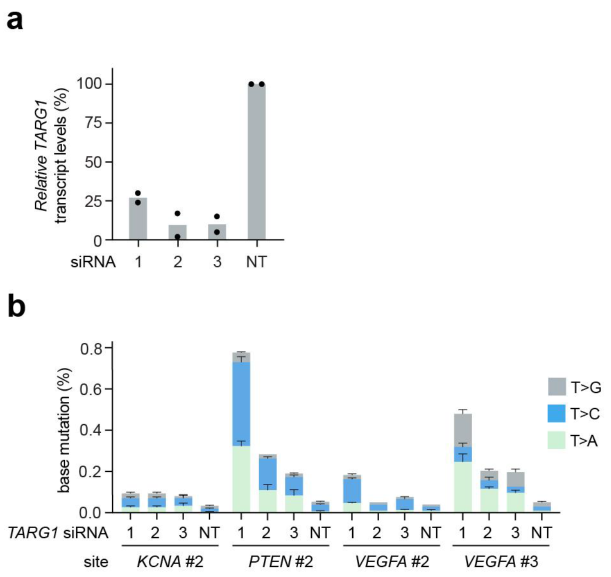

Supplementary Fig. 12: Base substitution frequencies via ADP-ribosylation in HEK293T cells subjected to siRNA-mediated silencing of *TARG1* expression. a, Reduction in *TARG1* transcript levels following siRNA treatment. Measurements were performed 72 hours post-transfection of the indicated siRNA. Quantified levels were normalized to that of the non-targeting (NT) siRNA conducted in the same experiments. Each dot represents a single independent experiment. **b,** Editing frequencies at the thymine ADP-ribosylated by DarT2^{D}-nSpCas9 is shown. Three different TARG1-targeting siRNAs were tested in comparison to the NT siRNA. Bars and error bars in a and b represent the mean and SEM of three independent replicates without selection or sorting.

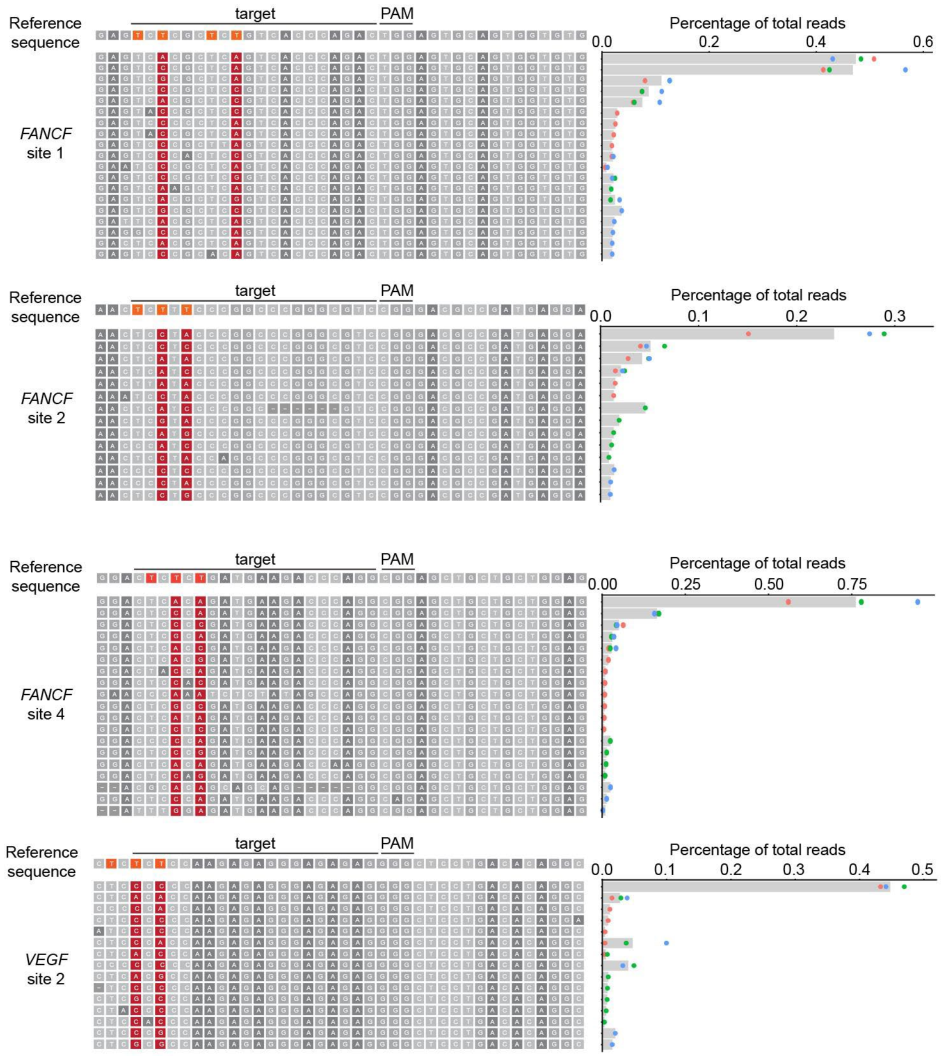

Supplementary Fig. 13: Frequency of co-occurring by-stander mutations when multiple motifs are present in the target sequence. Data analysed from targets *FANCF*_sg1, *FANCF*_sg2, *FANCF*_sg4 and *VEGFA*_sg2 in Figure 4e and Supplementary Fig. 11. Values reported represent the mean and SEM of three independent replicates without selection or sorting. Dots represent measurements from individual replicates.

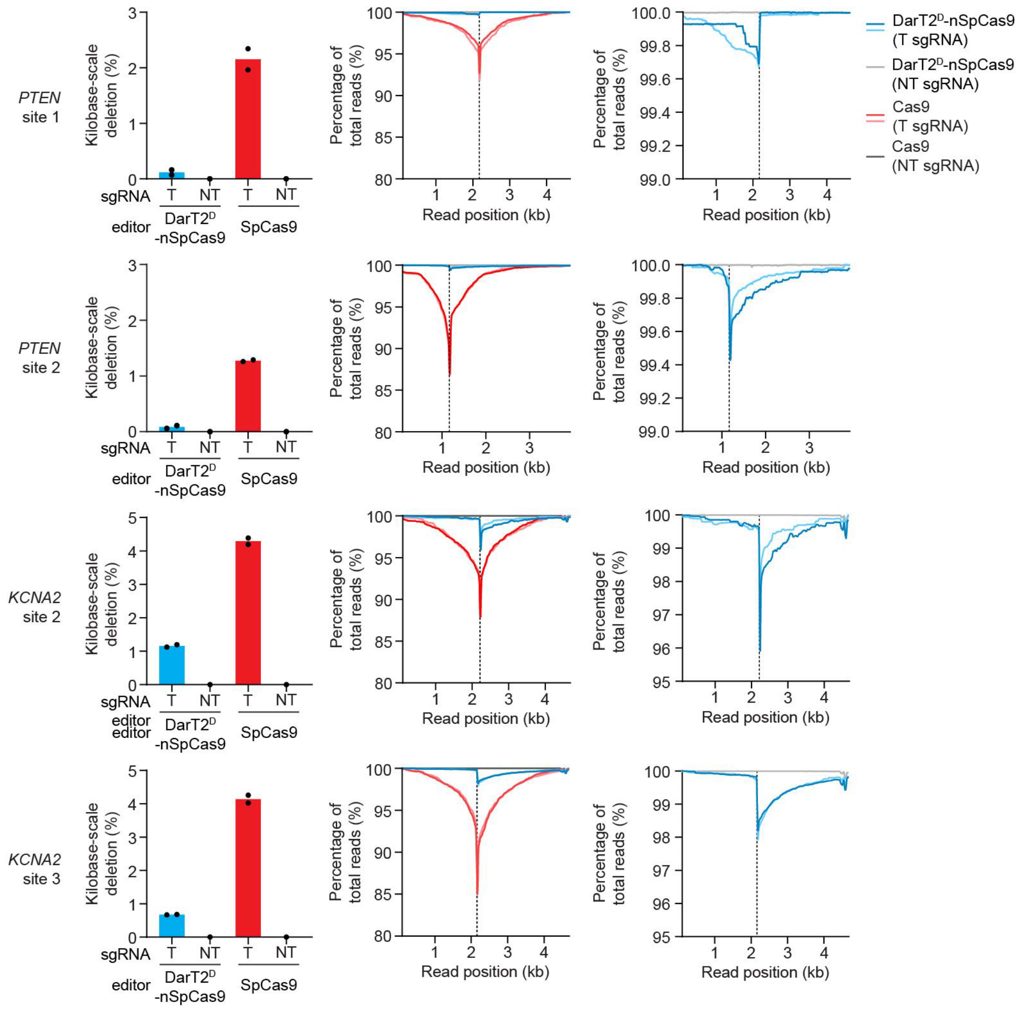

Supplementary Fig. 14: Programmable DNA ADP-ribosylation leads to limited large deletions. Kilobase-scale deletions were assessed at four sites undergoing thymine ADP-ribosylation by DarT2D-nSpCas9 or DNA cleavage by SpCas9. Left: frequency of large deletions. Bars represent the average of two independent experiments (targeting conditions) or one individual measurement (non-targeting conditions). Middle and right: distribution of deletions around the target site (dotted line). Lines with different shades of the same color represent replicates. T sgRNA, targeting sgRNA. NT sgRNA, non-targeting sgRNA.

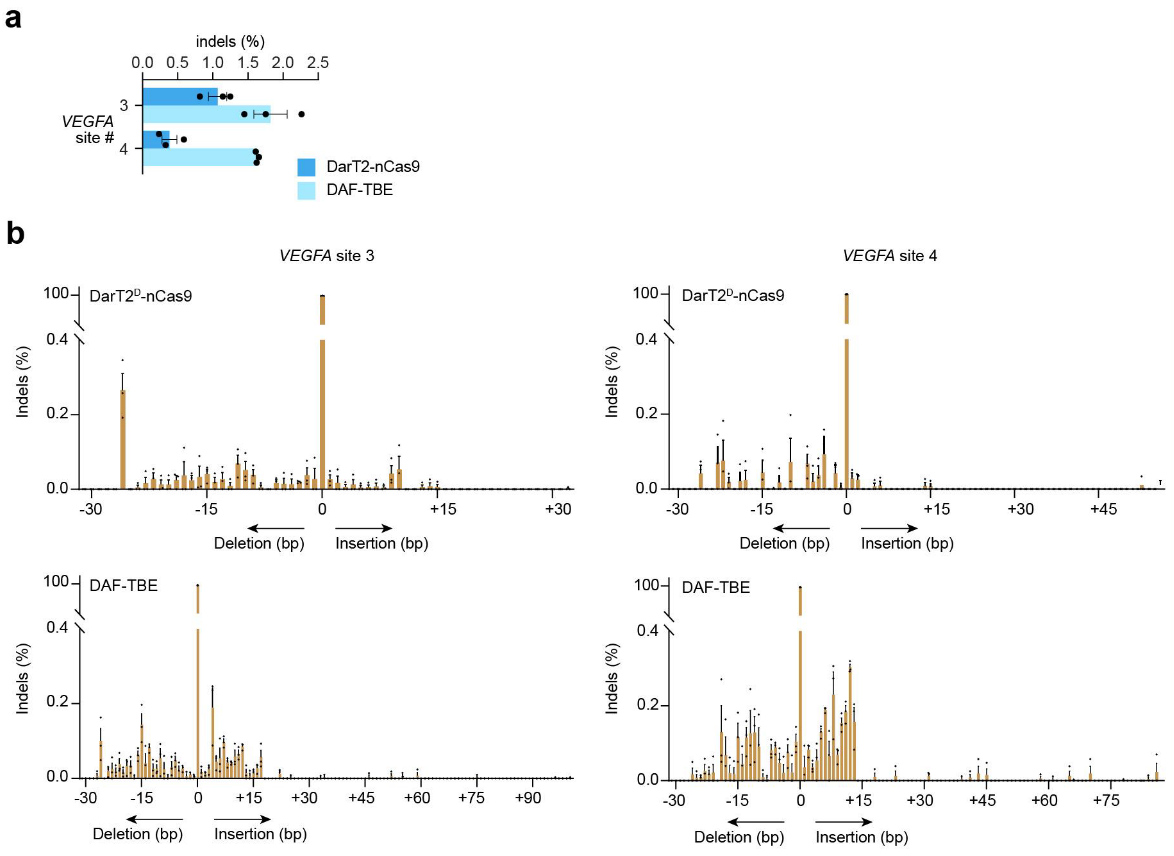

Supplementary Fig. 15: Indels formation in HEK293T Δ*TARG1* cells when targeting the same sites with DarT2^{D}-nSpCas9 and DAF-TBE. a, Indel frequencies. Indel formation was significantly lower at site 4 for DarT2D-nSpCas9 and statistically indistinguishable at site 3. Bars and error bars represent the mean and SEM of three independent replicates without selection or sorting. **b,** Distribution of indels for a representative experiment. Values with '-' reflect size of deletions, '+' reflect size of insertions, while '0' represents the percentage of reads containing no insertions or deletions. Bars and error bars represent the mean and SEM of three independent replicates without selection or sorting. The total indel frequency is shown in the upper-right.

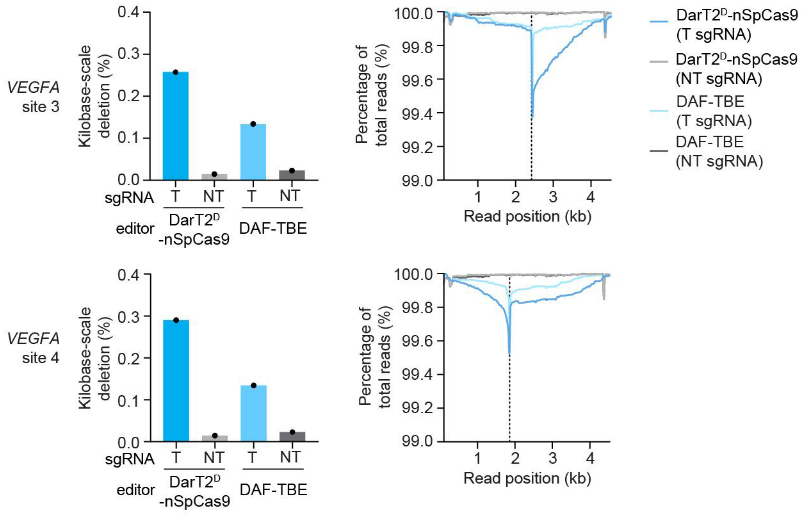

Supplementary Fig. 16: Large-scale deletions in HEK293T Δ*TARG1* cells when targeting the same sites with DarT2^{D}-nSpCas9 and DAF-TBE. Left: frequency of large deletions. Bars represent individual measurements. Right: distribution of deletions around the target site (dotted line). Lines with different shades of the same color represent replicates. T sgRNA, targeting sgRNA. NT sgRNA, non-targeting sgRNA.

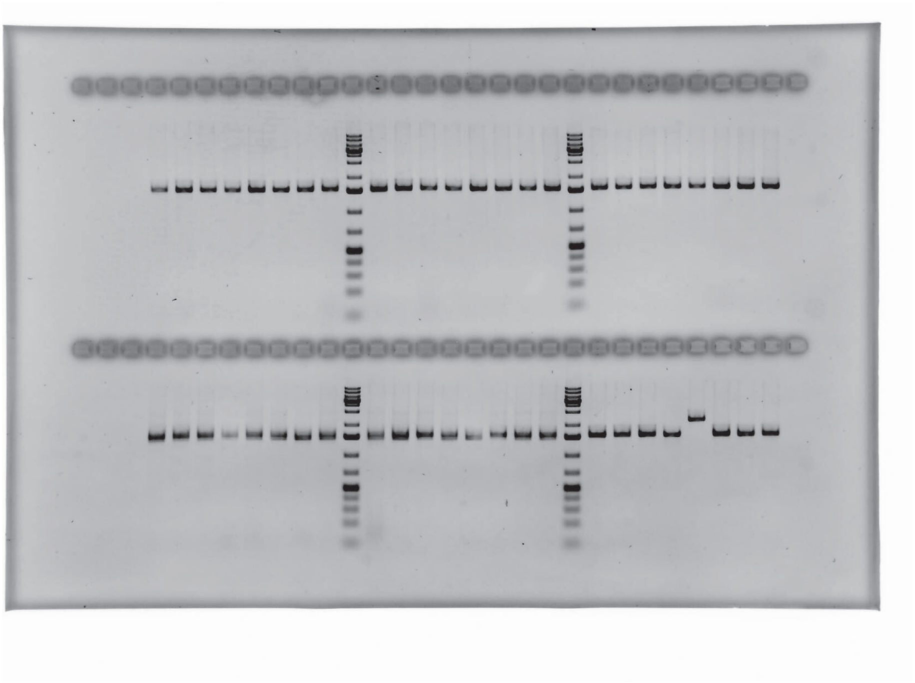

Supplementary Fig. 17: Uncropped image for agarose gel displayed in Supplementary Figure 4

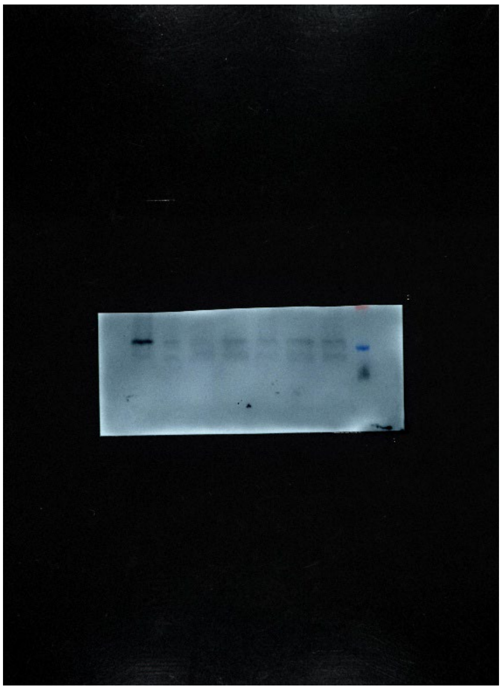

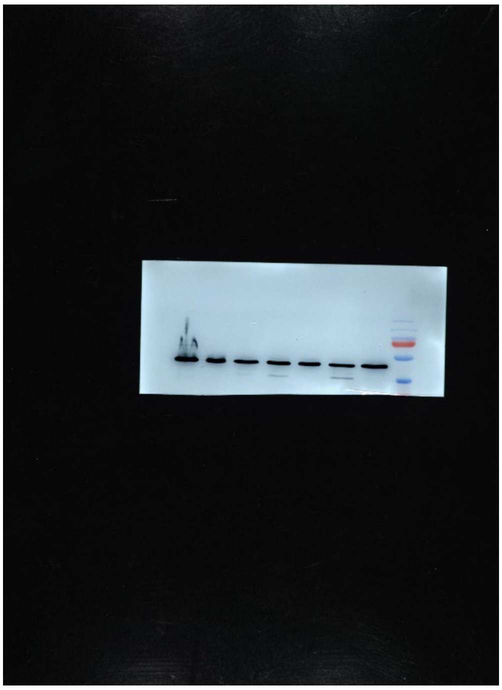

Supplementary Fig. 18: Uncropped images for western blot displayed in Supplementary Figure 8

SUPPLEMENTARY TABLES

Supplementary Table 1: Genome wide profiling of off-target single-nucleotide variations (SNVs) detected in the *kanR strain under non-targeting conditions.** Values correspond to Figure 1i. SNVs were re-coded to the strand containing an edited C or T. Position three in the motif corresponds to the Genomic Position at which the SNV was detected. Genomic positions correspond to *E. coli* MG1655 (GenBank: U00096.3).

Editor	SNV	Motif	Colony	Genomic Position	Read Depth	Percent
nCas9	C>G	GTCG	2	2884186	41	32
nCas9	C>G	TCCA	3	2496515	40	28
nCas9	C>G	GTCG	3	2884186	26	27
nCas9	C>G	CTCA	3	4002022	37	35
nCas9	C>T	ATCG	1	2238816	75	39
nCas9	T>C	ATTG	1	1728463	76	28
nCas9	T>C	AGTG	1	4032832	93	26
nCas9	T>C	GCTG	2	1964854	83	27
nCas9	T>C	ATTG	3	1996218	48	25
DarT(G49D)-nCas9	C>G	TCCA	1	2496515	57	26
DarT(G49D)-nCas9	C>G	CTCA	2	4002022	33	27
DarT(G49D)-nCas9	C>G	GTCG	3	2884186	29	31
DarT(G49D)-nCas9	C>T	ATCG	1	2238816	81	37
DarT(G49D)-nCas9	T>A	TTTC	1	1741177	71	55
DarT(G49D)-nCas9	T>C	TTTC	1	522283	58	48
DarT(G49D)-nCas9	T>C	TGTG	2	4413038	52	25
APOBEC-nCas9-UGI	C>T	CTCT	1	1632710	58	45
APOBEC-nCas9-UGI	C>T	CTCT	1	2555285	137	47
APOBEC-nCas9-UGI	C>T	CTCC	1	2557310	127	46
APOBEC-nCas9-UGI	C>T	CTCC	2	237578	221	33
APOBEC-nCas9-UGI	C>T	GTCC	2	250724	461	37
APOBEC-nCas9-UGI	C>T	TTCC	2	373332	508	53
APOBEC-nCas9-UGI	C>T	GTCA	2	437138	513	51
APOBEC-nCas9-UGI	C>T	GTCG	2	1602770	464	32
APOBEC-nCas9-UGI	C>T	TTCA	2	2737123	484	56
APOBEC-nCas9-UGI	C>T	CGCC	2	3543825	498	98

APOBEC-nCas9-UGI	C>T	ATCG	2	3561968	484	29
APOBEC-nCas9-UGI	C>T	TTCC	3	568237	251	33
APOBEC-nCas9-UGI	C>T	TTCC	3	569581	199	34
APOBEC-nCas9-UGI	C>T	TTCC	3	2189448	338	44
APOBEC-nCas9-UGI	T>C	TGTG	1	730239	77	26
APOBEC-nCas9-UGI	T>C	ATTG	1	3904655	116	29
APOBEC-nCas9-UGI	T>C	GCTG	1	4243748	124	26
APOBEC-nCas9-UGI	T>C	AATG	2	505166	483	25
APOBEC-nCas9-UGI	T>C	AATG	3	505166	440	25

Supplementary Table 2: Strains, plasmids, oligos, sgRNA guides and siRNAs used in this work. See the included Excel file.

Supplementary Table 3: Potential disease targets for ADPr-TAE. See the included Excel file.

Supplementary Table 4: Source data for Supplementary figures. See the included Excel file.

SUPPLEMENTARY REFERENCES

1. Tromans-Coia, C. *et al.* TARG1 protects against toxic DNA ADP-ribosylation. *Nucleic Acids Res.* **49**, 10477–10492 (2021).
2. Doench, J. G. *et al.* Optimized sgRNA design to maximize activity and minimize off-target effects of CRISPR-Cas9. *Nat. Biotechnol.* **34**, 184–191 (2016).
