# Supplementary material for: Targeted DNA ADP-ribosylation triggers templated repair in bacteria and base mutagenesis in eukaryotes
Source: Nat Biotechnol. 2025 Sep 4;44(7):1190–201. doi: 10.1038/s41587-025-02802-w (PMC13368585; doi:10.1038/s41587-025-02802-w)
Supplement: Supplementary file 2 — Reporting Summary [file 41587_2025_2802_MOESM2_ESM.pdf]

nature portfolio

Corresponding author(s): Prof. Chase Beisel

Last updated by author(s): Jul 4, 2025

Reporting Summary

Nature Portfolio wishes to improve the reproducibility of the work that we publish. This form provides structure for consistency and transparency in reporting. For further information on Nature Portfolio policies, see our Editorial Policies and the Editorial Policy Checklist.

Statistics

For all statistical analyses, confirm that the following items are present in the figure legend, table legend, main text, or Methods section.

n/a	Confirmed	
☐	☒	The exact sample size (*n*) for each experimental group/condition, given as a discrete number and unit of measurement
☐	☒	A statement on whether measurements were taken from distinct samples or whether the same sample was measured repeatedly
☐	☒	The statistical test(s) used AND whether they are one- or two-sided<br>*Only common tests should be described solely by name; describe more complex techniques in the Methods section.*
☒	☐	A description of all covariates tested
☐	☒	A description of any assumptions or corrections, such as tests of normality and adjustment for multiple comparisons
☐	☒	A full description of the statistical parameters including central tendency (e.g. means) or other basic estimates (e.g. regression coefficient) AND variation (e.g. standard deviation) or associated estimates of uncertainty (e.g. confidence intervals)
☐	☒	For null hypothesis testing, the test statistic (e.g. *F*, *t*, *r*) with confidence intervals, effect sizes, degrees of freedom and *P* value noted<br>*Give P values as exact values whenever suitable.*
☒	☐	For Bayesian analysis, information on the choice of priors and Markov chain Monte Carlo settings
☒	☐	For hierarchical and complex designs, identification of the appropriate level for tests and full reporting of outcomes
☐	☒	Estimates of effect sizes (e.g. Cohen's *d*, Pearson's *r*), indicating how they were calculated

Our web collection on statistics for biologists contains articles on many of the points above.

Software and code

Policy information about availability of computer code

Data collection	For bacterial and yeast experiments, Sanger sequencing was performed by Microsynth Seqlab GmbH. RTq-PCR was performed on a CFX96 Real-Time PCR Detection System (Bio-Rad). Nanopore sequencing was performed using services from Plasmidsaurus and Eurofins.
Data analysis	General analysis and plotting with Graphpad prism 10 software (version 10.4.1). Sanger sequence alignment was performed using Benchling. Whole genome alignments of E. coli were performed using Minimap2 (v2.28), alignment file processing used samtools (v1.16.1) and bcftools (v1.16) and variant calling was performed using Clair3 (v1.0.10).<br>For mammalian experiments, Illumina NovaSeqXSeries Control Software 1.2.2.48004 and NextSeq 2000 DRAGEN ORA system software was used for base-calling, for demultiplexing bcl-convert Version 4.2.7 was used. For analyzing editing outcomes, CRISPResso2 (https://crispresso2.pinellolab.org/submission) was used. Base-calling was performed using MinKNOW (v 24.11.11) for large scale deletions, summarizeOntDeletions (https://github.com/cornlab/summarizeOntDeletions) was used for quantification of deletions and BamCoverage was used from deeptools v3.5.6 for coverage plots.<br>For plant experiments, iSeq 100 Real Time Analysis (RTA v2) software was used for base-calling. Demultiplexing for the iSeq100 was performed using Local Run Manager (version 4.2.0.14) with the GenerateFASTQ workflow (version 2.5.56.27).<br>Custom R scripts were used for the analysis of processed NGS data (https://github.com/saliba-lab/ADPr_TAE_analysis) for mammalian cells and plants

For manuscripts utilizing custom algorithms or software that are central to the research but not yet described in published literature, software must be made available to editors and reviewers. We strongly encourage code deposition in a community repository (e.g. GitHub). See the Nature Portfolio guidelines for submitting code & software for further information.

Data

Policy information about availability of data

All manuscripts must include a data availability statement. This statement should provide the following information, where applicable:

- Accession codes, unique identifiers, or web links for publicly available datasets
- A description of any restrictions on data availability
- For clinical datasets or third party data, please ensure that the statement adheres to our policy

High-throughput sequencing data were submitted to Sequence Read Archive (SRA) and are available under the Bioproject number PRJNA1149814. Source data for all figures are provided in Table S2 and Table S3. There are no restrictions on data availability. Further information and requests for resources and reagents should be directed to and will be fulfilled by the lead contact (C.L.B., chase.beisel@helmholtz-hiri.de).

Research involving human participants, their data, or biological material

Policy information about studies with human participants or human data. See also policy information about sex, gender (identity/presentation), and sexual orientation and race, ethnicity and racism.

Reporting on sex and gender	None since using in vitro, bacterial, yeast, plants and common immortalized cell lines.
Reporting on race, ethnicity, or other socially relevant groupings	None since using in vitro, bacterial, yeast, plants and common immortalized cell lines.
Population characteristics	None since using in vitro, bacterial, yeast, plants and common immortalized cell lines.
Recruitment	None since using in vitro, bacterial, yeast, plants and common immortalized cell lines.
Ethics oversight	None since using in vitro, bacterial, yeast, plants and common immortalized cell lines.

Note that full information on the approval of the study protocol must also be provided in the manuscript.

Field-specific reporting

Please select the one below that is the best fit for your research. If you are not sure, read the appropriate sections before making your selection.

☒ Life sciences ☐ Behavioural & social sciences ☐ Ecological, evolutionary & environmental sciences

For a reference copy of the document with all sections, see nature.com/documents/nr-reporting-summary-flat.pdf

Life sciences study design

All studies must disclose on these points even when the disclosure is negative.

Sample size	For bacterial and yeast experiments, three independent transformation events were considered as biological replicates. 8 colonies from each resulting transformation were subjected to Sanger sequencing. In total, 24 colonies were screened through Sanger sequencing. For all cell based experiments, three independent replicates were used, as is the standard sample size for statistically significant biological tests. High throughput sequencing data was analyzed for three independent replicates and in some cases for two replicates, as mentioned within the respective legend. This is in line with the standard protocols in the field. This sample size is sufficient for the statistical analysis using tests mentioned under Methods. Detailed information about the sample numbers for each experiment is provided in the manuscript.
Data exclusions	All measured replicates are reported with no exclusions.
Replication	The reproducibility for the bacterial and yeast experiments was confirmed by the chosen number of biological replicates (3) and the screened resulting colonies from each replicate (8), totaling 24 colonies screened per experiment. The reproducibility of mammalian and plant experiments was confirmed with 3 replicates tested for all high throughput sequencing experiments unless noted otherwise.
Randomization	Randomization was not performed following standard procedures for molecular biology research.
Blinding	Blinding was not performed following standard procedures for molecular biology research.

Reporting for specific materials, systems and methods

We require information from authors about some types of materials, experimental systems and methods used in many studies. Here, indicate whether each material, system or method listed is relevant to your study. If you are not sure if a list item applies to your research, read the appropriate section before selecting a response.

Materials & experimental systems

n/a	Involved in the study
☐	☒ Antibodies
☐	☒ Eukaryotic cell lines
☒	☐ Palaeontology and archaeology
☒	☐ Animals and other organisms
☒	☐ Clinical data
☒	☐ Dual use research of concern
☐	☒ Plants

Methods

n/a	Involved in the study
☒	☐ ChIP-seq
☒	☐ Flow cytometry
☒	☐ MRI-based neuroimaging

Antibodies

Antibodies used	Anti-TARG1 antibody (Fisher Scientific, Cat. # 25249-1-AP)<br>Anti-beta-actin antibody (Life technologies, Cat. # MA5-15739-HRP)
Validation	The Anti-TARG1 antibody was validated in the reference: https://doi.org/10.1093/nar/gkab771, and by the manufacturer (https://www.thermofisher.com/antibody/product/C6orf130-Antibody-Polyclonal/25249-1-AP)<br>The Anti-beta-actin antibody was validated by the manufacturer (https://www.thermofisher.com/antibody/product/beta-Actin-Antibody-clone-BA3R-Monoclonal/MA5-15739-HRP) and in multiple references mentioned on the same page.<br>These were further tested in controls as part of this study.

Eukaryotic cell lines

Policy information about cell lines and Sex and Gender in Research

Cell line source(s)	The yeast strain Saccharomyces cerevisiae BY4741 with a genotype MATa his3Δ1 leu2Δ0 met15Δ0 ura3Δ0 was used. BY4741 is part of the EUROSCARF deletion collection, often used in functional genomics and systems biology studies<br>HEK293T cells were purchased from ATCC (CRL 11268) and U2OSΔTARG1 cell lines were a gift from the Ivan Ahel lab
Authentication	Cell lines were not authenticated in house.
Mycoplasma contamination	All cell lines negative for mycoplasma contamination (checked via PCR with primers specific for mycoplasma)
Commonly misidentified lines<br>(See ICLAC register)	No commonly misidentified cell lines used

Dual use research of concern

Policy information about dual use research of concern

Hazards

Could the accidental, deliberate or reckless misuse of agents or technologies generated in the work, or the application of information presented in the manuscript, pose a threat to:

No	Yes
☒	☐ Public health
☒	☐ National security
☒	☐ Crops and/or livestock
☒	☐ Ecosystems
☒	☐ Any other significant area

Experiments of concern

Does the work involve any of these experiments of concern:

No	Yes	
☒	☐	Demonstrate how to render a vaccine ineffective
☒	☐	Confer resistance to therapeutically useful antibiotics or antiviral agents
☒	☐	Enhance the virulence of a pathogen or render a nonpathogen virulent
☒	☐	Increase transmissibility of a pathogen
☒	☐	Alter the host range of a pathogen
☒	☐	Enable evasion of diagnostic/detection modalities
☒	☐	Enable the weaponization of a biological agent or toxin
☒	☐	Any other potentially harmful combination of experiments and agents

Plants

Seed stocks	Standard Nicotiana benthamiana wild-type
Novel plant genotypes	No new genotype was developed. Only transient expression experiments were carried out.<br>Transient expression was carried out using the Agrobacterium tumefaciens strain GV3101.
Authentication	Amplicon sequencing was used to confirm the identity of the N. benthamiana plants.
