# Supplementary figures and images for: Targeted DNA ADP-ribosylation triggers templated repair in bacteria and base mutagenesis in eukaryotes
Source: Nat Biotechnol. 2025 Sep 4;44(7):1190–201. doi: 10.1038/s41587-025-02802-w (PMC13368585; doi:10.1038/s41587-025-02802-w)

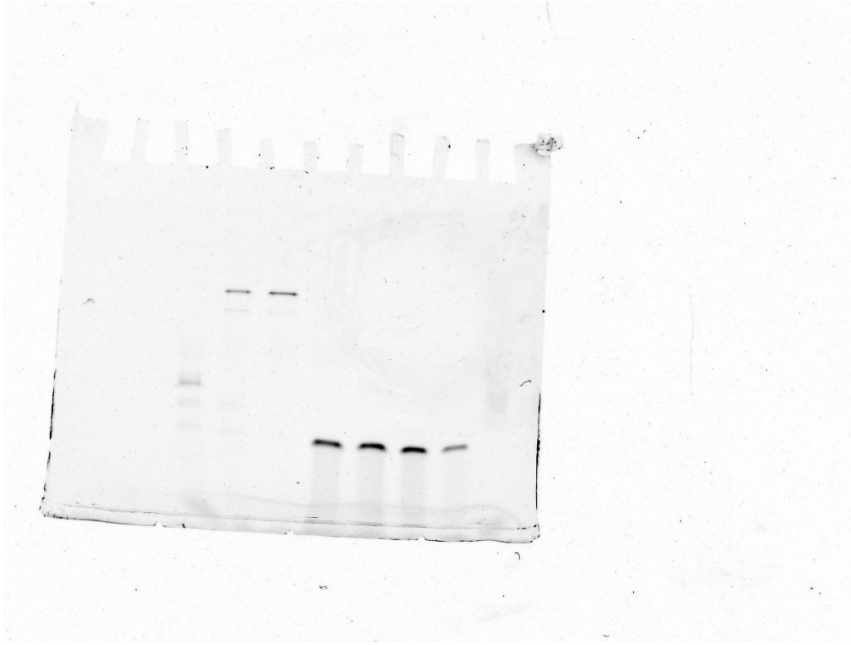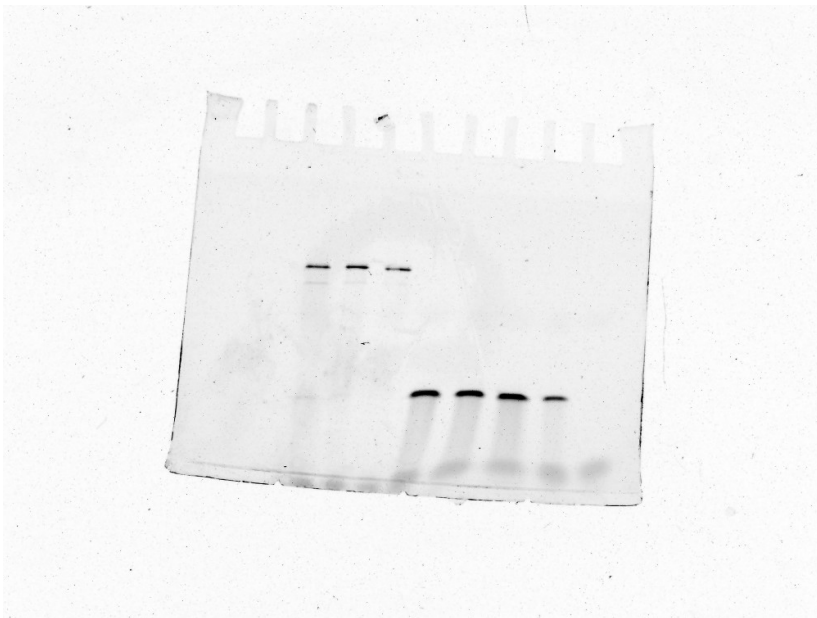

Uncropped images for gels displayed in **Figure 1d**.

Supplement: Supplementary file 7 — Unmodified gels. [file 41587_2025_2802_MOESM7_ESM.pdf]
